# Evidencing general acceptability of open-label placebo use for tackling overtreatment in primary care: a mixed methods study

**DOI:** 10.1186/s12916-023-03074-4

**Published:** 2023-09-19

**Authors:** E. M. Krockow, T. Emerson, E. Youssef, S. Scott, S. Tromans

**Affiliations:** 1 School of Psychology and Vision Sciences, University of Leicester, Leicester, UK; 2 School of Sociology and Social Policy, University of Nottingham, Nottingham, UK; 3 School of Nursing, Kingston University, London, UK; 4 School of Healthcare, University of Leicester, Leicester, UK; 5 Department of Population Health Sciences, University of Leicester, Leicester, UK; 6 Adult Learning Disability Service, Leicestershire Partnership NHS Trust, Leicester, UK

**Keywords:** Placebos, Open-label placebos, Overtreatment, Overprescribing, Antibiotics, Antidepressants, Analgesics, Antimicrobial resistance, Antimicrobial stewardship, Primary care

## Abstract

**Background:**

Overtreatment poses a challenge to healthcare systems due to harmful consequences of avoidable side-effects and costs. This study presents the first account for examining the feasibility of placebo use for reducing overtreatment in primary care, including whether public attitudes support the use of different placebo types in place of inappropriate prescriptions of antibiotics, antidepressants, or analgesics.

**Methods:**

We used a multi-study, mixed-methods design, including patient and public (PPI) consultations, focus groups (Study 1) and two pre-registered online experiments (Studies 2 and 3).

**Results:**

Study 1 (*N* = 16) explored everyday conceptions and practicalities of potential placebo use in the context of respiratory infections. Findings highlighted the importance of trusting doctor-patient relationships and safety-netting. Study 2 employed a randomised experiment with a representative UK sample (*N* = 980), investigating attitudes towards 5 different treatment options for respiratory infections: (1) blinded + pure placebo, (2) open-label + pure placebo, (3) open-label + impure placebo, (4) antibiotic treatment, and (5) no treatment. Study 2 also examined how attitudes varied based on wording and individual differences. Findings indicated general support (*η*_p_^2^ = .149, large effect size) for replacing inappropriate antibiotics with open-label + impure placebos, although personal placebo acceptability was lower. Also, older people, individuals suffering from chronic illness or those showing higher levels of health anxiety appeared less amenable to placebo use. Study 3 (*N* = 1177) compared attitudes towards treatment options across three clinical scenarios: respiratory infection, depression and pain. Findings suggested significant differences in the acceptability of placebo options based on the clinical context. In the infection scenario, options for open-label + pure placebos, open-label + impure placebos and no treatment were rated significantly more acceptable (*η*_p_^2^ = .116, medium effect size) compared to the depression and pain scenarios. Again, general support for placebos was higher than placebo acceptability for personal use.

**Conclusions:**

Findings from PPI and three studies indicate general support for combatting overprescribing in primary care through clinical placebo use. This is an indicator for wider UK public support for a novel, behavioural strategy to target a long-standing healthcare challenge. General acceptability appears to be highest for the use of open-label + impure placebos in the context of antibiotic overprescribing.

**Supplementary Information:**

The online version contains supplementary material available at 10.1186/s12916-023-03074-4.

## Background

This article presents the first comprehensive evidence around public acceptability of clinical placebo use as a behavioural strategy to reduce overtreatment in “primary care”, defined by the World Health Organisation as “a model of care that supports first-contact, accessible, continuous, comprehensive and coordinated person-focused care” [1]. Overtreatment, referring to the unnecessary use of medicines with questionable patient benefits, poses a significant challenge to modern healthcare systems due to harmful consequences of avoidable side effects, wasteful use of resources and unnecessary economic costs [2]. Especially in the context of increasing healthcare burdens to the NHS, there is increased recognition of the need to tackle overtreatment (e.g. the BMJ’s Too Much Medicine initiative [3] and the NHS England Stopping Overmedication of People with a Learning Disability, Autism, or Both [STOMP] initiative [4].

While reasons for medicine overuse vary, key factors include diagnostic uncertainty and defensive medicine, aimed to reduce chances of patient litigation [5]. Behavioural scientists have also noted the importance of related cognitive biases, referring to systematic deviations from rational judgements, which may affect clinical diagnoses and subsequent treatment approaches [6, 7]. A cognitive bias repeatedly highlighted in the context of medicine overuse is the so-called action bias [6, 8–10], which refers to an inherent human preference of active problem solutions over passive alternatives, even in situations where active solutions are unlikely to yield superior results [11]. In healthcare, action bias may manifest itself through patient or prescriber preferences for active forms of treatment such as the immediate use of medicines as opposed to more passive approaches including watchful waiting and symptom monitoring.

A theory-based, behavioural solution to action bias is substitution [6], which describes a strategy of substituting undesirable actions (e.g. unnecessary use of potentially harmful and/or costly medication) with more desirable actions (e.g. use of harmless alternatives). This article explores the feasibility of substituting undesirable actions of overtreatment in primary care with the use of different placebo types.

### Placebo use in primary care

Placebos are “inert” substances that have no therapeutic effects but can alleviate symptoms through patients’ participation in the therapeutic encounter and the associated, measurable effects on neurobiological mechanisms [12]. Traditionally, placebo use has been limited to clinical trials of new medicines, where placebo use served as a control condition [13]. However, an emerging body of research considers clinical use of placebos as a form of treatment in its own right. While placebos do not treat the underlying organic cause of a disease in the same way as surgery or medication, they have the potential to produce measurable symptom improvement for a wide range of conditions [12].

Placebo types can be categorised based on the amount of information provided upon administration. Most historical placebo use has included some level of deception, meaning that patients were misled into believing that the prescribed placebo substances constituted a type of medical treatment [12]. To limit ethical concerns associated with the use of such “deceptive” or “blinded” placebos, recent research has trialled the use of so-called “open-label placebos”, which involves complete transparency about the nature of the substance administered. A systematic review of 11 clinical trials involving open-label placebo use suggests that even if patients know they are taking placebos, they still experience symptom relief for various conditions (e.g. back pain, cancer-related fatigue and ADHD) [14]. A potential reason for the surprising effectiveness may be the patients’ expectations associated with the mere action of taking a prescribed substance and their overall experience of a clinical treatment environment [15].

An additional distinction of placebos categories is made based on the type of substance administered [16, 17]. “Pure” placebos describe substances like sugar pills that look exactly like a form of active medication but do not have any pharmacologically active ingredients. “Impure” placebos, on the other hand, are replacement treatments that offer some symptom relief but do not treat the primary illness. Examples include conventional medicine with little evidence base (e.g. anti-inflammatory lozenges to relieve common cold symptoms) or nutritional supplements (e.g. vitamin pills to treat cancer). Notably, our working definition of impure placebos is closely aligned with a definition by Fent et al. [17]: “Impure placebos have pharmacological effects, but the effect on the specific disease the substance is prescribed for has not been proven or is uncertain.” (p.2), but differs in its wording from a previous definition by Howick et al.’s [16]: “substances, interventions or “therapeutic” methods which have known pharmacological, clinical or physical value for some ailments but lack specific therapeutic effects or value for the condition for which they have been prescribed” (p. 2). We chose to simplify this Howick et al.’s definition for the benefit of lay audiences and believe that it still retains key aspects of impure placebos including a focus on physical value (e.g. symptom relief) without curing the underlying condition.

Informal, clinical use of placebos is prevalent across different global healthcare contexts, but quantification of use is complicated due to varying definitions of impure placebos [18]. A 2013 survey attempted to measure the frequency of placebo treatments in UK primary care and findings indicated that the majority of UK General Practitioners (GPs) used some form of placebo treatment in their practice, often motivated by the wish to satisfy patient requests for active medical treatment [16]. However, the context and extent of UK placebo use appeared highly varied. 97% of surveyed GPs reported previous use of impure placebos (e.g. vitamin supplements for cancer treatment, probiotics for diarrhoea or peppermint pills for pharyngitis), while only 12% reported previous use of pure placebos (e.g. saline injections).

### Public acceptability of placebo use

The present research aims to test clinical placebo use as a substitution strategy to reduce the overuse of unnecessary medicines in UK primary care. Based on decision theory and empirical results pertaining to placebo effectiveness, we predict the benefits of placebos to be twofold. Firstly, they offer a behavioural solution to action bias that may satisfy both doctors’ and patients’ desires for an active treatment solution while reducing the use of potentially harmful medicines. Secondly, they are likely to provide significant symptom relief without reliance on actual medical treatment.

Given the evidence for existing non-systematic placebo use in UK primary care, there is a strong rationale for formalising placebo use by investigating conditions for successful clinical use across specific disease contexts. However, a key factor influencing the feasibility of any wider placebo roll-out is public acceptance of different placebo types as well as related beliefs about their efficacy.

Studies of placebo health literacy show that most members of the general public have a satisfactory but narrow working definition of placebos as “sham treatments” [19], that require a degree of deception. Initial research on public attitudes around placebos provides encouraging results, suggesting that patients are generally willing [19–22] and curious [23] to try to different types of placebos, which is often driven by a pragmatic desire to find something that successfully improves symptoms [24]. Qualitative studies highlighted the importance of trust in doctor-patient relationships as a prerequisite for placebo acceptability [23, 25, 26]. Furthermore, while participants typically expressed greater doubts about the efficacy of open-label placebos [23], many had a preference for full information about the prescribed substance [24].

Previous research shares a number of key limitations, meaning that there is no reliable evidence on public acceptability of clinical placebo use for tackling overtreatment. Firstly, many studies relied on small sample sizes and/or samples from specific cultural contexts [19, 20, 23, 25, 26]. Also, a lot of research was conducted alongside clinical trials and failed to consider the unique context of clinical placebo use [23, 25]. The few studies investigating acceptability of routine placebo use in clinical practice typically did not explicitly explore placebo use as a strategy for reducing overtreatment. For example, a survey conducted in New Zealand included much more varied reasons for placebo use (e.g. cases where doctors suspected patients pretending to be sick) [20]. One recent quantitative study specifically tested placebo use as a means to reducing antibiotic overprescribing in hospitals, but the research did not specify exact criteria for placebo use in their scenarios. Instead, participants rated their acceptance of giving hospital doctors a blanket permission to use blinded pure placebos at their own discretion [21]. Finally, no study to date included a controlled, experimental comparison of all different types of placebos highlighted in the introduction. Many studies focused narrowly on blinded pure placebos only [20], while others compared the acceptability of blinded and open-label placebos, without considering impure variants [25] or while failing to control for confounding variables. For example, UK focus groups explored attitudes towards a range of different placebos, but the hypothetical treatment scenarios varied in their ethical contexts (e.g. comparing self-limiting viral infections with terminal illness) [24].

In addition to these general limitations, there are a number of factors that have received comparatively little attention despite their theoretical relevance to the topic; these include the potential importance of language and framing and the influence of individual difference variables on placebo acceptability.

#### Framing effects

Evidence from the decision sciences shows that the specific way information is worded or “framed” can influence the way information is processed, perceived and subsequently acted upon. For example, a classic study suggested that framing the same treatment outcomes either in terms of losses (number of patients lost) or gains (number of patients saved) affected people’s acceptance of the treatment programme in question [27]. Previous research further argued that disease or medication names might influence the way the general public judge different health threats [28, 29]. Little attention has been paid to the use of language in the context of placebos. Some clinical trials used the term “sugar pill” as a synonymous term for “placebo” in patient communications [30, 31]. Another, less common variant was the term “dose extender” [32, 33]. To date it is unclear if or how specific terminology could affect placebo attitudes of lay populations, but it is possible that less scientific-sounding terminology (e.g. sugar pills) may reduce patient beliefs around placebo efficacy.

#### Individual differences

A 2020 systematic review suggests that personality variables may affect the strength of patients’ placebo responses, with optimism leading to higher effectiveness of placebos and health anxiety leading patients to experience more negative side effects (“nocebo effects”) when taking placebos [34]. Furthermore, related research on medical maximising and minimising suggests that individual differences exist with regard to preferences for more versus less healthcare [35]. Some individuals may prefer receiving less medicines, which may coincide with stronger support for the use of placebos. Nevertheless, there appears to be a lack of research that considers the effect of personality on attitudes and beliefs towards placebos. Health anxiety and health-related risk aversion might predict people’s risk perceptions of receiving placebos and their subsequent acceptance levels of clinical placebo use. Additionally, a person’s health literacy (i.e. their competence to process and assess health information) [36], which was found to predict the ability to interpret health messages and adhere to prescriptions [37], might predict whether individuals believe in the efficacy of placebos or find them acceptable for use in clinical practice.

### Research aims

This study aimed to test the feasibility of a novel, behavioural approach for tackling the international healthcare challenge of overtreatment in primary care. Specifically, we set out to provide the first comprehensive, mixed-method investigation of UK public attitudes towards different types of placebos and their potential clinical use to reduce overprescribing. Three studies addressed the following objectives:

- *Objective 1*: Explore everyday conceptions and practicalities of potential clinical placebo use through in-depth qualitative methods (Study 1).
- *Objective 2*: Experimentally test acceptability and beliefs of efficacy pertaining to the use of different placebo types for replacing inappropriate antibiotic prescriptions in the context of infection, while investigating influences of terminology and demographic covariates (Study 2).
- *Objective 3*: Test generalisability of placebo attitudes by comparing acceptability and efficacy ratings across three common contexts of overprescribing in primary care; infection, depression and chronic pain (Study 3).

## Study 1: focus groups

The first study consisted of an in-depth qualitative exploration of public attitudes around placebos. Focus groups were employed to generate discussion between different participants. At the same time, this initial qualitative study served to test the wording and general appropriateness of the patient scenario for later use in our follow-up experiments. Study 1 focused on exploring potential placebo use in the context of respiratory infections, where overprescribing of antibiotics is a very common problem [38]. This clinical context was chosen due to the frequency of overtreatment and the severity of its consequences, which includes the contribution to the global health emergency of antimicrobial resistance (AMR), as well as patient side effects.

### Methods

#### Participants

Using advertisements on social media (Twitter, Facebook), we recruited an opportunity sample of 16 adult members of the general public living in the UK. The sample included 9 males and 7 females with a mean age of 26.56 (*SD* = 3.45) years. The sample was ethnically diverse; 2 participants identified as being of “white” race, while the rest identified as “black/African/Caribbean/black British or other”. A detailed overview of demographic participant details is provided in Additional File 1: Table 1. Each participant was remunerated with a £20 shopping voucher.

#### Materials and procedure

The project idea and research plans including draft focus group materials were presented at two separate PPI (Patient and Public Involvement) group meetings of the “Leicester, Leicestershire and Rutland Ageing Patient and Public Involvement Forum” and the “Ethnic Minority Research Inclusion (EMRI) Hub (South Yorkshire)” prior to data collection. Overall, PPI group members were supportive of the research ideas, but highlighted a number of potential limitations and practical barriers to clinical placebo use, which were considered by the research team and implemented in the study materials. One PPI group suggestion included asking focus groups about the acceptability of charging patients for placebo prescriptions. A separate question on this topic was included in the topic guide.

Four virtual focus groups were conducted 2–4 November 2022 and lasted 30–70 min. Group sizes depended on participant availability and turn-up rates. Groups 1–4 comprised four, three, two and seven participants, respectively. All participants were recruited through an advertisement on social media. Prior to participation, they completed a consent form and a pre-study questionnaire including demographic items and basic questions about existing knowledge or previous experiences with placebos. According to this questionnaire, all participants had a personal working definition of placebos, but no participant was an expert in the topic (e.g. a medical prescriber or researcher in the field). All focus groups were conducted by the same researcher (EMK), following the same semi-structured topic guide (see Additional File 2). The guide included an introduction and explanation of the house rules (e.g. mutual respect and equal opportunities to engage). Then, the researcher gave a short presentation of key definitions for the different types of placebos and asked participants about their general attitudes and beliefs pertaining to placebo efficacy. Subsequently, the researcher presented a hypothetical patient vignette, which involved a patient presenting with a respiratory infection that shared some symptoms of bacterial infections (i.e. raised temperature), but overall failed to meet the NICE FeverPAIN criteria for the prescription of antibiotics [39]. The scenario included situational indicators that stressed the urgency of recovery (financial pressures and the need to return to work) and stated the patient’s explicit wish for treatment. At the same time, the scenario mentioned that unnecessary antibiotics could incur avoidable side effects. Participants were asked to discuss the acceptability and expected efficacy of placebo prescriptions as a substitute for inappropriate antibiotic use in the scenario. After completion of the topic guide, any additional comments were invited from participants before the researcher closed the focus group.

#### Data analysis

Focus group recordings were transcribed verbatim and imported into NVivo 12 for coding. In accordance with a Grounded Theory approach [40], the analysis followed an iterative process in which the data was initially open coded, the emergent findings discussed by the research team and grouped into provisional axial categories, and then further revised, compared and reduced upon additional rounds of coding in order to discover theory from the data.

### Results

The responses from the focus groups fell into three primary categories: ethical considerations around placebo usage, perceptual and conceptual understandings of placebos by the respondents, and practicalities of using placebos within healthcare provision. A detailed overview of individual codes as well as exemplary participant quotations is provided in Table 1.

**Table 1:** Axial codes, individual codes and example quotations for the qualitative focus group results

Axial codes:	Codes:	Example quote:
Ethical considerations	Trust and understanding	“…if it’s for something that is, I don’t know, like managing pain, if my GP gave me a placebo and I found out it was like a blinded placebo, I think I would have been annoyed and I think it potentially could undermine my relationship with my practitioner. So I would say it depends on how you use it and for what you use it” (FG4, P10)
	Deontologicalism vs. utilitarianism	“…if you are prescribing in medical practice a blinded placebo, I think that’s really ethically problematic because you are lying. Well, not lying. You are, you are not giving the full information to patients. […]you are also asking GPs not to give the full information to their patients. So, I think it’s, it’s quite hard on both sides of the dynamic. If you’re going to […] replace a conventional treatment with a placebo.” (FG1, P1)
	Contexts for use	“…as I mentioned like viral infections, you can’t treat with antibiotics because that only works for like bacterial infections. So I think in this case it’s OK to give, well in my opinion, it’s OK to give a placebo because antibiotics wouldn’t do anything for this particular case. And monitoring the patient to make sure it isn’t bacterial is like fine.” (FG4, P10)
Perceptions and conceptions of placebos	Terminology	“They don’t work, but they do help and there is a semantic definition between those two things, and I think that’s really important to keep in mind. Like, they don’t work. They absolutely don’t do anything, but they do help. They do, you know, provide support. They do. And, and whilst those two things I think can sometimes get conflated, I think for me, it feels very important to remember that they are quite separate.” (FG1, P1)
	Conceptualising existing actions and substances as placebos	“…perhaps there is some form of like learned response that our body produces. So, for example, I don’t want to take decaf coffee […] almost like my body feels like that it is actual coffee because of the association I have with how it looks and all that stuff…” (FG4, P10)
	A “non-real” treatment	“OK to me. I think a placebo is, you know, is designed to seem like a real treatment, but it’s, it’s not real and it doesn’t have a, an actual effect on the condition for the purpose of, of the treatment. And I think it also based on, you know, a kind of positive thinking that is like psychology.” (FG2, P6)
	Attitudes around placebos	“I feel the mindset of, of the person taking this placebo is also considered. If you go with the mindset of what I’m taking is going to work for me, then definitely it’s going to work for you. But if you just taking it like, ok, this is what was administered to me. I just have to take it then then the probability of it working it’s let’s say it’s a 50–50 thing. So, it has to do with your mindset, your conviction, what you believe in. (FG4, P12)
Practicalities of placebo use	Practicalities of use	“For me, looking at this placebo, it didn’t fall from the sky. So if I give it to you, you have to pay for it. So he’s, he’s gonna pay for it.” (FG4, P13)
	Wider ramifications of use	“This is the other thing is that if you start prescribing these things and giving them out for free, it’s quite likely you will see an uptake in people going to their GP for things that they don’t need to go to a GP for. And that has its own impact in terms of people not being able to either get a GP appointment, things they do need. Or to GP’s just being overwhelmed with loads and loads of patients, who really, they don’t need to see.” (FG1, P1)
	Individual physiological considerations	“…we have different body systems and the way our body responds to most of these medications, they differ. So what might what work in your body system not really work for me. So, we really need to check out how our system responds to this treatment […] to know if this placebo that’s been administered to us has been effective or not.” (FG4, P13)

#### Theme 1: ethical considerations

Trust within the patient-clinician relationship was viewed as foundational to the use of placebos in appropriate conditions and contexts. Primarily, trusting that a clinician would be using the placebo for an appropriate condition—i.e. one that would not be worsened by substituting a placebo—and in a context that was beneficial for the patient. Participants suggested increasing levels of societal enculturation to build trust, via public health campaigns and clinician-based education during consultations. However, some participants expressed concern that the offer of a placebo could undermine the seriousness a patient had attached to their complaint, and erode the relationship and trust between the patient and clinician, especially in vulnerable communities with low health adherence. Participants also considered the ethical concerns of deontological and utilitarian aspects of placebo use. They generally preferred open-label placebos to the inherently deceptive use of blind placebos, which they perceived as violating the professional duties of care by not keeping the patient fully informed—regardless of being potentially more efficacious. Participants identified specific circumstances where placebo use could be ethically acceptable, such as mental health contexts, while pain alleviation was viewed as an unsuitable application. The expected temporality of the illness played a role in how ethically suitable placebo use was perceived, with short-term applications for minor acute illnesses viewed as relatively low risk, while long-term use of placebos was seen as high risk for a patient.

#### Theme 2: perceptions and conceptions of placebos

The discussions also revealed how participants perceived placebos as a “non-real” form of treatment, with descriptors such as “pseudo-medication”, “does nothing”, and “not a real treatment” being used. Nevertheless, the placebo effect itself was largely accepted and spoken about as a real phenomenon. Participants suggested that the terminology around how placebos functioned should be clear to patients, indicating that placebos “help” or provide temporary “relief” rather than “work”. The labels “pure” and “impure” were also viewed as holding inherent connotations that could potentially affect a patient’s attitude towards placebo use. Some participants likened their perception of placebos to foods in which an active ingredient has been removed (e.g. decaffeinated coffee) or to lifestyle cosmetics that have unproven health benefits but create a “feel good” mindset through the action of attending to health.

Participants noted that a patient's initial mindset or expectations prior to receiving a placebo could expedite their efficacy. Initial positive expectations were linked to a more widespread enculturation around the use of placebos as a legitimate form of treatment, and the role of clinicians in engendering a positive view of placebo use for their patients. A patient’s religious or socio-cultural background was viewed as potentially influencing their perceptions towards placebos and their predisposition towards their use; alongside previous poor experiences with healthcare services.

#### Theme 3: practicalities of placebo usage in healthcare

Participants debated whether placebos should be priced as regular medication, with most believing that as a tangible good or service received from a healthcare provider, a placebo should be treated monetarily similar to any other medication. However, others viewed charging for placebos as either unethical or unfair to patients. Some suggested alternative funding routes for placebos through charities or subsidisation. Individual physiological differences between patients and the degree to which this affected receptiveness were raised as a potential practical barrier to the widespread use of placebos as substitutes. Participants also considered wider ramifications, such as the potential reinforcement of a transactional, medicalized response to ailments, increased traffic towards primary care services, and the potential spread of infection within primary care sites.

### Brief discussion

Our study aligns with previous research indicating trust in doctor-patient relationships as crucial for placebo utilisation [23–26] and the ethical debate surrounding consequentialism/utilitarianism and deontologicalism as a vital question over their potential use [24]. Our findings also support prior literature suggesting the public views placebos as a “non-real” treatment and prefer open-label placebos [19, 24]. The participants’ engagement in scenarios suggesting placebo use for minor respiratory infections exemplified these findings. Although our participants understood the open/closed label distinction (informing the use of this terminology in Studies 2 and 3), the reported connotations in the pure/impure designation led to the avoidance of these terms in our subsequent quantitative studies.

The participants’ identification of mental health contexts as suitable for placebo use suggests a promising avenue for further research; especially when this is contrasted with their view of placebo use as unsuitable for tangible conditions like chronic pain and considered alongside the generally held view of placebos as a “non-real” medication. Based on these findings, we decided to include scenarios involving mental health problems and chronic pain in Study 3.

## Study 2: experimental test of placebo acceptability and framing effects in the context of antibiotic overprescribing

A nationally representative UK sample was recruited to increase the generalisability of our qualitative findings and experimentally test acceptability and efficacy ratings for different placebo treatments to substitute unnecessary antibiotic use in primary care. Additionally, we compared two framing contexts, while also testing for the potential role of demographic factors and individual difference variables.

### Hypotheses

Our quantitative hypotheses were based on previous research findings and included the following:

1. Qualitative hypothesis: Existing perceptions of placebos will vary with regard to the underlying level of knowledge and personal attitudes. However, a common theme will be a perceived need for deception to ensure placebo effectiveness.
2. Framing hypothesis: Participant ratings for general treatment acceptability, personal treatment acceptability and expected treatment efficacy of different placebos will be affected by the terminology used. Participants in the sugar pill condition (vs. placebo condition) will rate placebo treatments as more acceptable, but less effective.
3. Treatment type hypothesis: There will be significant differences in the acceptability and effectiveness ratings for different treatment options in the hypothetical patient scenarios. Open-label placebos will be rated more acceptable but less effective than blinded placebos. Any placebo treatment will be rated as less acceptable and less effective compared to antibiotic treatment. Acceptability and effectiveness ratings will be lowest for the “no treatment” scenario.
4. Demographic hypothesis: Participants of a lower age (vs higher age), and participants, who have visited a doctor in the past 12 months (vs. not visited a doctor), taken antibiotics in the past 12 months (vs. not taken antibiotics), never taken a placebo (vs. previously taken a placebo) and who consider themselves to have a chronic illness or be immunocompromised (vs. healthy with normal immune systems) will rate placebo treatments as more acceptable and effective.
5. Covariate hypothesis: High scores in health anxiety and health risk aversion and low scores in health risk literacy will be correlated with lower acceptance levels and lower effectiveness ratings of placebos. All three variables will explain a significant amount of variance in the ANCOVA analysis.

### Methods

#### Participants

Following an a priori power calculation with an estimated a power of 0.99 and an α error probability of 0.01 to detect a medium effect size *f* = 0.25., we recruited a nationally representative sample of 1000 participants via the online recruitment platform *Prolific* in November 2022. Each participant received a remuneration of £1.50 (£9.00 pro-rata). 20 participants were excluded from the study because they did not meet the screening criteria (see Data analysis section). The final participant sample had a mean age of 46.49 years (*SD* = 15.84) and included 470 males, 505 females, 1 individual identifying as ‘other’ and 4 individuals preferring not to disclose the information. Additional demographic information is provided in Additional File 1: Table 2.

#### Design

We employed a 2 × 5 between-within subjects design and measured three dependent variables. This included (1) the general acceptability of a treatment offered in a hypothetical patient scenario (measured on a 5-point Likert scale ranging from “highly unacceptable” to “highly acceptable”), (2) the personal acceptability of a treatment offered in a hypothetical patient scenario (measured on 5-point Likert scale ranging from “very unhappy to receive this treatment” to “very happy to receive this treatment”), and (3) expected effectiveness of a treatment offered in a hypothetical patient scenario (measured on 5-point Likert scale ranging from “very ineffective” to “very effective”). Participants were randomly assigned to one of two between-subjects framing groups (“placebo” vs. “sugar pill”), which determined the wording of treatment options in the patient scenario they were shown. All participants completed five within-subjects treatment conditions in randomised order, including (1) blinded + pure placebo, (2) open-label + pure placebo, (3) open-label + impure placebo, (4) antibiotic treatment, and (5) no treatment. In the “sugar pill” framing group, the term “placebo” was consistently replaced with the term “sugar pill”.

Additionally, we assessed three individual difference variables: (1) health anxiety (Likert rating ranging from 1 to 4, with low scores indicating low health anxiety), (2) health risk literacy (Likert rating ranging from 1 to 3, with low scores indicating low health risk literacy) and (3) health risk aversion (Likert rating ranging from 1 to 5, with low scores indication low risk-seeking). Finally, we tested the impact of several quasi-experimental between-subject variables including sex (female/male) and age (high/low) and four medical history variables: “doctor’s visits over the past year” (yes/no), “antibiotics used during the past year” (yes/no), “previous use of placebos” (yes/no) and “affected by chronic illness and/or an immunocompromised state” (yes/no).

#### Materials and procedure

Study completion took approximately 10 min. The mean completion times were 13:34 min for Study 1 and 11:05 min for Study 2. Participants completed the study in their own time and could decide for themselves how much time to spend on each section. All materials can be accessed in Additional File 2. The study was hosted on the online platform *Gorilla* and consisted of four parts. After giving informed consent, participants completed Part 1, which included questionnaires assessing demographic variables and relevant medical history (e.g. previous experience with placebos). Part 2 included three open-ended questions asking participants to provide a definition of placebos/sugar pills as well as the advantages and disadvantages of their use. Part 3 consisted of a hypothetical patient scenario involving a respiratory infection that was almost identical to the one used in Study 1. It only differed in the name and age of the fictitious patient and the amount of detail given about the patient’s circumstances. Also, in contrast to the scenario in Study 1, Study 2 mentioned the potential increase of drug-resistant bacteria as a consequence of antibiotic overuse. The scenario was followed by a presentation of five treatment options in random order, involving different placebo types, antibiotics or no treatment at all. The impure placebo option included anti-inflammatory lozenges as an example of medicine without evidence base [41]. For each treatment option, participants were asked to rate general acceptability, personal acceptability and expected effectiveness. Finally, Part 4 included three psychometric questionnaires to assess health anxiety (Health Anxiety Questionnaire [42]), health risk literacy (All Aspects of Health Literacy Scale [43]) and health risk aversion (DOSPERT Risk-Taking Scale, Health/Safety Sub-Scale [44]).

#### Data analysis

All data were rigorously vetted prior to the analyses. We excluded participants who met one or more of the following criteria: (1) identical responses to all three qualitative questions, (2) study completion in less than 5 min (which may indicate a lack of engagement with the study contents) or more than 60 min (which may indicate a high level of distraction during study completion), (3) suspected of “straightlining” (i.e. providing the same answers to all quantitative questions). Criteria for straightlining were set as having a standard deviation of ≤ 0.4 for the Likert ratings across all five treatment scenarios and/or a standard deviation of 0 for all Likert responses to the psychometric questionnaires.

##### Qualitative analyses

The free-text responses to the survey were examined using content analysis—a systematic method for uncovering patterns and themes within qualitative data [45, 46]. The data underwent an iterative coding process in which an initial reading was made, preliminary coding categories assigned and then refined and revised on further analysis to arrive at coherent themes from the data.

##### Quantitative analyses

Before conducting any ANCOVAs or ANOVAs, assumptions were tested for each DV by checking for significant outliers, producing QQ plots and running Shapiro–Wilk tests (to check normality assumptions), running Mauchly’s test of sphericity, conducting Levene’s tests for homogeneity of variance and running a Box’s M test to check for homogeneity of covariances. Additionally, we produced scatterplots to check for linear relationships between the covariates and each DV and ran tests for homogeneity of regression slopes. Most assumptions were met, but the results showed the existence of some outliers and violations of the normality and sphericity assumptions across almost all conditions of all DVs. Additionally, there were violations of the assumptions regarding linearity and homogeneity of regression slopes for covariates. Given our large sample size, we considered ANOVAs robust to normality violations. To account for violations of sphericity, we will report Greenhouse–Geisser adjusted values throughout. However, given the assumption violations pertaining to our covariates, we decided against conducting an ANCOVA. Instead, we transformed the continuous covariate scores for age, health anxiety (HAQ), health literacy (HLQ) & and health risk-taking (DOSPERT) into binary variables, in order to enter these as independent variables in additional ANOVA analyses. This was done by summing individual participant ratings for the respective questionnaire items. For the health literacy questionnaire, items 1 and 3 were reverse coded. Subsequently, the median score was determined and participants were re-coded as either high- or low-scoring depending on their score’s position relative to the median. Participants with a HAQ score of > 34 were coded as “high in health anxiety” (vs. low in health anxiety), participants with an HLQ score of > 25 were coded as “high in health literacy (vs. low in health literacy) and participants with a DOSPERT score of > 10 were coded as “high in health risk taking” (vs. low in health risk taking). Similarly, demographic variables were transformed into binary variables. Participants aged > 46 years were recoded as “high age” (vs. “low age). For analyses involving the variable “sex”, participants were excluded if they identified as “other” (*N* = 1) or preferred not to disclose their sex (*N* = 4).

### Results

#### Content analysis of qualitative answers

An overview of content codes and associated frequencies is provided in Additional File 1: Table 3. The respondents’ definitions of placebos were primarily focused on their inert properties and the notion that they represent a type of fake or artificial medication. In addition, there was a significant emphasis on the idea that the use of placebos in healthcare would involve deception, as well as the psychological mechanisms that were deemed essential for their efficacy, such as belief. Notably, several responses defined placebos in the contexts of research or clinical trials—indicating a perception that placebos are not intended for use in routine clinical practice.

The participants identified positive psychological effects and the utility of placebos in monitoring medication efficacy as major advantages. In addition, respondents also highlighted the absence of side effects associated with placebos, as well as their potential to reduce the use of unnecessary medications, thus serving to reduce costs to both the patient and NHS.

However, the potential for patient deception and the fact that placebos are fundamentally inactive were the primary disadvantages identified by respondents. Relatedly, the participants highlighted that the use of placebos could delay the administration of necessary active medications, resulting in the prolongation or exacerbation of illness. Some respondents also proposed that the use of placebos could erode trust in clinicians and healthcare providers.

#### Effects of framing and treatment conditions on dependent variables

**Fig. 1:**
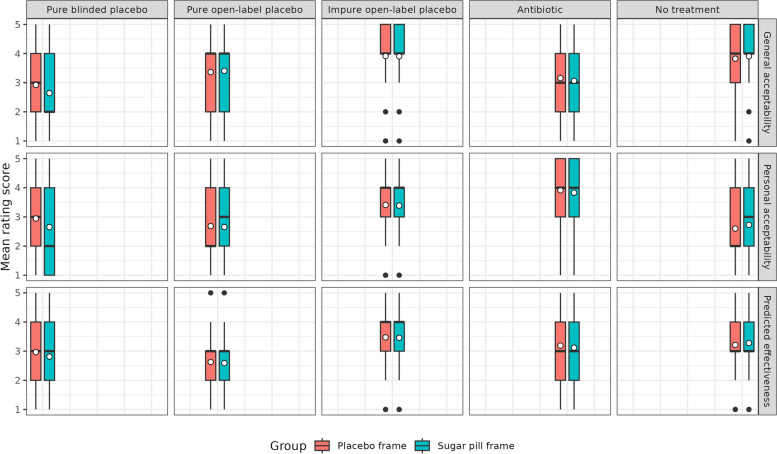
Study 2 ratings for general acceptability, personal acceptability and predicted effectiveness across five treatment conditions. Box plot showing the rating scores for general acceptability, personal acceptability and predicted effectiveness across the five treatment conditions (blinded + pure placebo; open-label + pure placebo; open-label + impure placebo; antibiotic treatment; no treatment) and two framing groups (placebo frame; sugar pill frame)

Three 2 × 5 mixed ANOVAs were conducted to test for effects of the framing group (placebo vs. sugar pill) and the treatment condition (blinded + pure placebo vs. open-label + pure placebo vs. open-label + impure placebo vs. antibiotic treatment vs. no treatment) on general acceptability, personal acceptability and expected effectiveness of the treatment. Descriptive statistics for all groups and conditions are presented in Additional File 1: Table 4. Figure 1 visualises the results with box plots.

For general acceptability, there was a significant interaction effect, *F*(3.161, 3091.25) = 3.791, *p* < 0.001, *η*_p_^2^ = 0.015, indicating that the rating of the treatment conditions differed depending on the framing group. Contrasts showed that the sugar pill frame produced a lower rating than the placebo frame for the “pure blinded placebo” condition. With regard to main effects, there was a non-significant main effect of the framing group (*p* > 0.05) and a significant main effect of the treatment condition, *F*(3.161, 3091.25) = 171.381, *p* < 0.001, *η*_p_^2^ = 0.149. Post hoc comparison using Bonferroni adjustments indicated that all treatment conditions produced significantly different mean ratings. The open-label + impure placebo condition received the highest ratings, and this was followed by the no treatment” condition, the open-label + pure placebo condition, the antibiotics condition and lastly the blinded + pure placebo condition.

For personal acceptability, there was a significant interaction effect, *F*(3.488, 3411.38) = 4.309, *p* < 0.01, *η*_p_^2^ = 0.004, again suggesting that the rating of the treatment conditions differed depending on the framing group. Contrasts showed that the sugar pill frame produced a lower rating than the placebo frame for the “blinded + pure placebo” condition. With regard to main effects, there was a non-significant main effect of the framing group (*p* > 0.05) and a significant main effect of the treatment condition, *F*(3.488, 3411.38) = 211.613, *p* < 0.001, *η*_p_^2^ = 0.178. Personal acceptability ratings were highest for the “antibiotics” condition, followed by “open-label + impure placebo”, “blinded + pure placebo”, “open-label + pure placebo”, and “no treatment”. Post hoc comparison showed that antibiotics and open-label impure placebos were rated significantly higher on personal acceptability than the other three conditions.

For expected effectiveness, there was no significant interaction effect. The main effect of the framing group was again non-significant (*p* > 0.05). However, we found a significant main effect of the treatment condition, *F*(3.309, 3235.97) = 103.923, *p* < 0.001, *η*_p_^2^ = 0.096. Post hoc comparison showed that all treatment conditions differed significantly from one another. The “open-label + impure placebo” condition was rated as the most effective, followed by “no treatment”, “antibiotics”, “blinded + pure placebo” and “open-label + pure placebo”.

#### Effects of demographic variables and medical history on dependent variables

Additional 2 × 5 mixed ANOVAs were conducted to test for effects of the binary variables pertaining to demographic information and medical history and the treatment conditions on general acceptability, personal acceptability and expected effectiveness of the treatment. Only significant results will be presented below. Full tables with means and standard deviations for all additional ANOVAs are included in Additional File 1:Tables 5–11.

##### Age

Age showed significant interactions with treatment conditions across all three DVs. For general acceptability (*F*(3.162, 3023.29) = 2.592, *p* < 0.05, *η*_p_^2^ = 0.003), people aged above 46 years (compared to those aged 46 years or below) provided lower ratings for open-label pure placebos. For personal acceptability (*F*(3.480, 3326.53) = 5.265, *p* < 0.01, *η*_p_^2^ = 0.005), those in the higher age category gave higher ratings for the no treatment option. Finally, for predicted effectiveness (*F*(3.305, 3159.87) = 4.671, *p* < 0.01, *η*_p_^2^ = 0.005), participants in the older age category gave significantly lower ratings for blinded pure and open-label pure placebos as well as antibiotic treatment. A significant main effect for age (*F*(1, 956) = 10.033, *p* < 0.01, *η*_p_^2^ = 0.01) suggested that participants of the older age category gave lower effectiveness ratings overall.

##### Sex

Sex showed significant interactions with treatment conditions across all three DVs. For general acceptability (*F*(3.151, 3.065.62) = 8.683, *p* < 0.001, *η*_p_^2^ = 0.009), women rated pure blinded placebos significantly lower than men. For personal acceptability (*F*(3.482, 3387.67) = 9.183, *p* < 0.001, *η*_p_^2^ = 0.009), women, again, rated pure blinded placebos significantly lower than men. Finally, for predicted effectiveness (*F*(3.305, 3215.55) = 4.135, *p* < 0.01, *η*_p_^2^ = 0.004), women gave significantly higher ratings for open-label pure placebos and the no treatment option, compared to men.

##### Antibiotics taken

Whether or not participants had taken antibiotics in the past 12 months had significant interaction effects with treatment conditions across all three DVs. Participants who had recently taken antibiotics, rated the antibiotics treatment option as more generally acceptable (*F*(3.211, 3136.90) = 20.457, *p* < 0.001, *η*_p_^2^ = 0.021), more personally acceptable (*F*(3.48, 3403.53) = 5.925, *p* < 0.001, *η*_p_^2^ = 0.006) and more effective (*F*(3.343, 3990.30) = 15.222, *p* < 0.001, *η*_p_^2^ = 0.015) than participants, who had not taken antibiotics.

##### Chronic illness and immunocompromised conditions

An underlying chronic illness or immunocompromised condition was found to have significant main effects on general treatment acceptability (*F*(1, 969) = 8.980, *p* < 0.01, *η*_p_^2^ = 0.009), and predicted treatment effectiveness (*F*(1, 969) = 6.426, *p* < 0.05, *η*_p_^2^ = 0.007), with participants suffering from such conditions giving lower mean ratings than other participants. For personal acceptability, a significant interaction effect between chronic illness and treatment condition was found (*F*(3.48, 3372.42) = 3.082, *p* < 0.05, *η*_p_^2^ = 0.003), with contrasts indicating that chronic illness patients found blinded pure and open-label pure placebos less personally acceptable and the no treatment option more personally acceptable compared to participants not suffering from a chronic illness or an immunocompromised condition.

#### Effects of individual differences on dependent variables.

Additional 2 × 5 mixed ANOVAs were conducted to test for effects of the binary variables pertaining to individual differences and the treatment conditions on general acceptability, personal acceptability and expected effectiveness of the treatment. Again, only significant results will be presented below.

##### Health anxiety

Health anxiety produced significant interaction effects with the treatment condition for general acceptability (*F*(3.179, 3109.45) = 5.991, *p* < 0.001, *η*_p_^2^ = 0.006) and predicted effectiveness (*F*(3.32, 3250.91) = 6.279, *p* < 0.001, *η*_p_^2^ = 0.006). Participants with higher health anxiety rated antibiotic treatment as more generally acceptable and more effective and no treatment as less generally acceptable and less effective compared to individuals with lower health anxiety. For personal acceptability, a significant main effect was found. Participants with higher health anxiety provided lower mean ratings for personal acceptability across all treatments (*F*(1, 978) = 4.599, *p* < 0.05, *η*_p_^2^ = 0.005).

##### Health literacy

Health literacy was found to have a significant interaction effect with the treatment condition on general acceptability (*F*(3.16, 3097.74) = 3.471, *p* < 0.05, *η*_p_^2^ = 0.004). Participants with higher health literacy gave lower mean ratings for the treatment options of blinded pure placebos and antibiotics.

##### Health risk-taking

Health-related risk-taking has a significant interaction effect with the treatment condition on personal acceptability (*F*(3.49, 3414.56) = 4.346, *p* < 0.005, *η*_p_^2^ = 0.004). Participants reporting higher levels of risk-taking found the option of no treatment less acceptable than participants with lower levels of risk-taking.

### Brief discussion

Study 2 provides the first comprehensive evidence of the general acceptability of clinical placebo use by the UK general population. Our nationally representative online study confirmed beliefs in efficacy and general support for the use of open-label impure placebos to replace unnecessary antibiotic treatments for respiratory infections in primary care. Below follows a detailed discussion of our specific hypotheses.

The content analysis of free text answers confirmed the qualitative hypothesis—the theme of deception featured highly in both the participants’ definitions of placebos, as well as in their identifications of disadvantages associated with placebo use. This was also echoed in the suggestion that a further disadvantage to placebo use would be the loss of trust in healthcare providers for potentially misleading patients.

Our framing hypothesis was partially confirmed. The framing group produced significant interaction effects, but the effects differed from our predictions. General and personal acceptability of blinded pure placebos were higher in the placebo group compared to the sugar pill group. Even though effect sizes were small, these findings appear to suggest that describing placebos as “sugar pills” could decrease people’s acceptability for clinical placebo use, specifically with regard to blinded pure placebo types.

Our treatment type hypothesis was partially confirmed. The treatment condition had significant effects on placebo attitudes in the hypothetical patient scenario, but the effects differed slightly from out predictions. Open-label impure placebos were rated highest in terms of general acceptability and predicted effectiveness, but scored slightly lower in terms of personal acceptability. Indeed, in terms of personal acceptability, participants showed a significant preference for antibiotic treatment. This suggests that even though the UK general public expressed general support for open-label placebo use, their attitudes differed if they imagined themselves to be the patients. These findings link in with patient attitudes in wider healthcare settings. For example, UK national surveys suggest that almost 90% of the population believe emergency care services are used inappropriately [47]. Yet, with visits to Emergency Departments on a rise again following the pandemic [48], it appears there is a mismatch between general attitudes about inappropriate health-seeking behaviours and people’s personal healthcare choices. Reasons for this mismatch between general and personal attitudes need to be better understood, but psychology research on self- and other decision-making offers initial explanations. Studies show that people often make different choices for themselves and others. Specifically, when choosing for themselves, people often exhibit a stronger focus on preventing negative outcomes and show a greater sensitivity to potential losses [49, 50]. This loss and risk aversion might also explain the somewhat contradictory finding that participants had a personal preference for antibiotic treatment even though open-label placebo use was generally rated as more effective. It is possible that “real medication” (i.e. antibiotics) was perceived as less effective but also less risky due to being the more established treatment option. In the context of placebo use, personal acceptability might therefore be improved through safety-netting designed to avoid negative outcomes (e.g. the scheduling of review appointments).

Our demographic hypothesis was again partially confirmed. Most of the demographic and medical history variables investigated had significant effects on placebo attitudes. As predicted, younger participants had more favourable attitudes towards placebo treatments compared to older participants. This might be explained by a greater openness of younger people towards new or alternative treatment options. Additionally, interesting sex differences were found, with female participants being more dismissive of blinded pure placebos and having stronger faith in the efficacy of open-label pure placebos compared to males. This seems to suggest that women are more sensitive to deception, supporting previous findings that women are more averse to lying than men [51, 52]. Testing the effects of previous antibiotic use, we found that participants who had taken antibiotics over the past 12 months provided higher ratings for the antibiotic treatment option. It is possible that recent positive experiences of antibiotic use prompted this comparatively stronger support for antibiotic medication. Finally, we found that participants suffering from chronic illness provided lower acceptability and effectiveness ratings across all treatment options and found placebo use particularly unacceptable for their personal circumstances, which might be explained by the higher risks involved in treating infections of patients with underlying conditions. Notably, while we report some significant findings pertaining to the medical history variables, it is difficult to draw final conclusions, because participant numbers reporting previous placebo use (*N* = 21), a chronic illness (*N* = 193) or recent antibiotic use (*N* = 265) or no recent visits to their doctors (*N* = 295) were comparatively small, resulting in very uneven group sizes for our between-subjects comparison.

Finally, our covariate hypothesis on the effects of health anxiety, health literacy and health-related risk-taking was partially confirmed. Health anxiety had significant effects on placebo attitudes. Higher anxiety levels resulted in higher acceptability of antibiotic use and lower acceptability of the no treatment option, which might be explained by higher levels of risk aversion leading to a preference of medicine treatments over placebo use. It is possible that anxious patients have stronger preferences for “real” medication and feel more apprehensive about less established treatment alternatives including placebos. Additionally, our findings suggested that patients with lower levels of health literacy may hold more favourable attitudes towards the use of antibiotics and blinded pure placebos. A potential reason for their antibiotic treatment preference could be lower awareness of the threat imposed by antimicrobial resistance. Their comparatively stronger acceptance of blinded pure placebos might be explained by lower levels of objection towards deception, but further qualitative investigations are needed to explore the reasons in detail.

Overall, our findings suggest generally favourable attitudes towards open-label placebo use to replace antibiotic medication that lacks a clear indication. However, our findings of different preferences when making personal treatment choices (as opposed to expressing more general attitudes) need to be addressed. The personal preference for antibiotic treatment could present a challenge when trying to implement clinical placebo use in practice. Given that this personal preference is likely to be fuelled by higher levels of personal risk and loss aversion, strategies of patient safety-netting (e.g. regular reviews post-placebo prescription) might provide reassurance and result in a greater personal willingness to forgo unnecessary prescriptions of active medicines. In any case, the use of placebo treatments may need to take into account individual patient characteristics. Our results suggest that older individuals with chronic illnesses or high levels of health anxiety might be less open to placebo prescriptions and that patients with lower health literacy may have a stronger preference for real medicines and blinded pure placebos, thus making them less accepting of open-label placebos specifically. Additionally, the use of blinded pure placebos appears to be particularly unacceptable to female individuals. To gain a more thorough understanding of attitudinal predictors and influences, follow-up qualitative investigations could be fruitfully employed.

## Study 3: experimental test of placebo acceptability across different contexts of primary care prescribing

Study 3 aimed to extend the previous quantitative study by comparing placebo attitudes across a wider range of clinical scenarios. In addition to the initial scenario of respiratory infections, we included vignettes involving less severe depression and lower back pain, where participants had to rate the acceptability and expected effectiveness of different placebo types compared with active medical treatment in the form of antidepressants and prescription pain killers, respectively. The scenarios of less severe depression and chronic back pain were chosen because they are contexts of frequent overtreatment in primary care [53, 54] and because focus group participants from Study 1 had made direct references to placebo use in mental health and pain management. Psychiatry, in particular, had been highlighted as a medical speciality that might be particularly suited to placebo use.

### Hypotheses

Our quantitative hypotheses were based on the findings from Study 1 and Study 2. The hypotheses reported and tested here deviate slightly from our pre-registration. We had planned to test, again, for the effects of demographic variables, medical history and individual differences variables on placebo attitudes. However, given the comprehensive analyses conducted as part of Study 2, we decided to exclusively focus on the comparison of different clinical scenarios and their effects on attitude ratings pertaining to different treatment options.

1. Clinical scenario hypothesis: Participant ratings for general treatment acceptability, personal treatment acceptability and expected treatment efficacy of different placebos will be affected by the clinical scenario presented. Participants in the infection condition (vs. depression and back pain conditions) will rate placebo treatments as less personally acceptable. Participants in the depression condition (vs infection condition and back pain conditions) will rate placebo treatments as more acceptable and effective.
2. Treatment type hypothesis: There will be significant differences in the acceptability and effectiveness ratings for different treatment options in the hypothetical patient scenarios. Open-label placebos (where participants are fully informed about their placebo treatment) will be rated more acceptable but less effective than blinded placebos. Impure placebos like lozenges/vitamin pills/heat spray, which are treatments with known pharmacological value for some of the symptoms, but without therapeutic effects for the underlying illness, will be rated as most acceptable and effective compared to all other placebo types. Any placebo treatment will be rated as less acceptable and less effective compared to the non-placebo (i.e. active medical) treatment.

### Methods

#### Participants

Following an a priori power calculation with an estimated a power of 0.99 and an α error probability of 0.01 to detect a medium effect size *f* = 0.25., we recruited a nationally representative sample of 1200 participants via the online recruitment platform Prolific in February 2023. Each participant received a remuneration of £1.50 (£9.00 pro-rata). 18 participants were excluded from the study because they did not meet the screening criteria (see Data analysis section). The final sample had a mean age of 45.93 years (*SD* = 15.49) and included 558 males, 616 females, and 3 individuals identifying as ‘other’. Additional demographic information is provided in Additional File 1: Table 12.

#### Design

We employed a 3 × 5 between-within subjects design and measured the same three dependent variables as in Study 2. Participants were randomly assigned to one of three between-subjects groups, which differed in the disease context of the patient scenario. Group 1 were presented with the same scenario of a respiratory infection involving potential antibiotic treatment as in Study 2. Group 2 were presented with a scenario of less severe depression involving potential treatment with antidepressants. Group 3 were presented with a scenario of lower back pain involving potential treatment with prescription pain killers. Like in Study 2, all participants completed five within-subjects treatment conditions in randomised order, including (1) blinded + pure placebo, (2) open-label + pure placebo, (3) open-label + impure placebo, (4) medicine treatment, and (5) no treatment.

#### Materials and procedure

Materials and procedure were almost identical to Study 2. The main difference pertained to the use of clinical scenarios. Instead of a single focus on infections, we included three alternative patient scenarios describing either a respiratory infection, a case of less severe depression or a case of chronic back pain (see full materials in Additional File 2). The examples given for open-label impure placebos included anti-inflammatory lozenges in the infection scenario, vitamin B12 pills in the depression scenario (as an example of a nutritional supplement [16]) and pain relief heat spray in the back pain scenario (as an example of a medicine without evidence base [55]). To avoid undue influences of confounding factors, scenarios were matched in length, wording and key situational aspects of the scenario (e.g. information highlighting the need of a fast recovery, the role of patient expectations, and the GP’s offer for review in case of placebo prescriptions).

#### Data Analysis

The same quality screening criteria were applied as in Study 2. Variables were computed the same way as in Study 2. Again, assumptions of normality and sphericity were violated for our ANOVAs, which is why we will report Greenhouse–Geisser adjustments throughout.

### Results

#### Effects of scenario group and treatment conditions on dependent variables

**Fig. 2:**
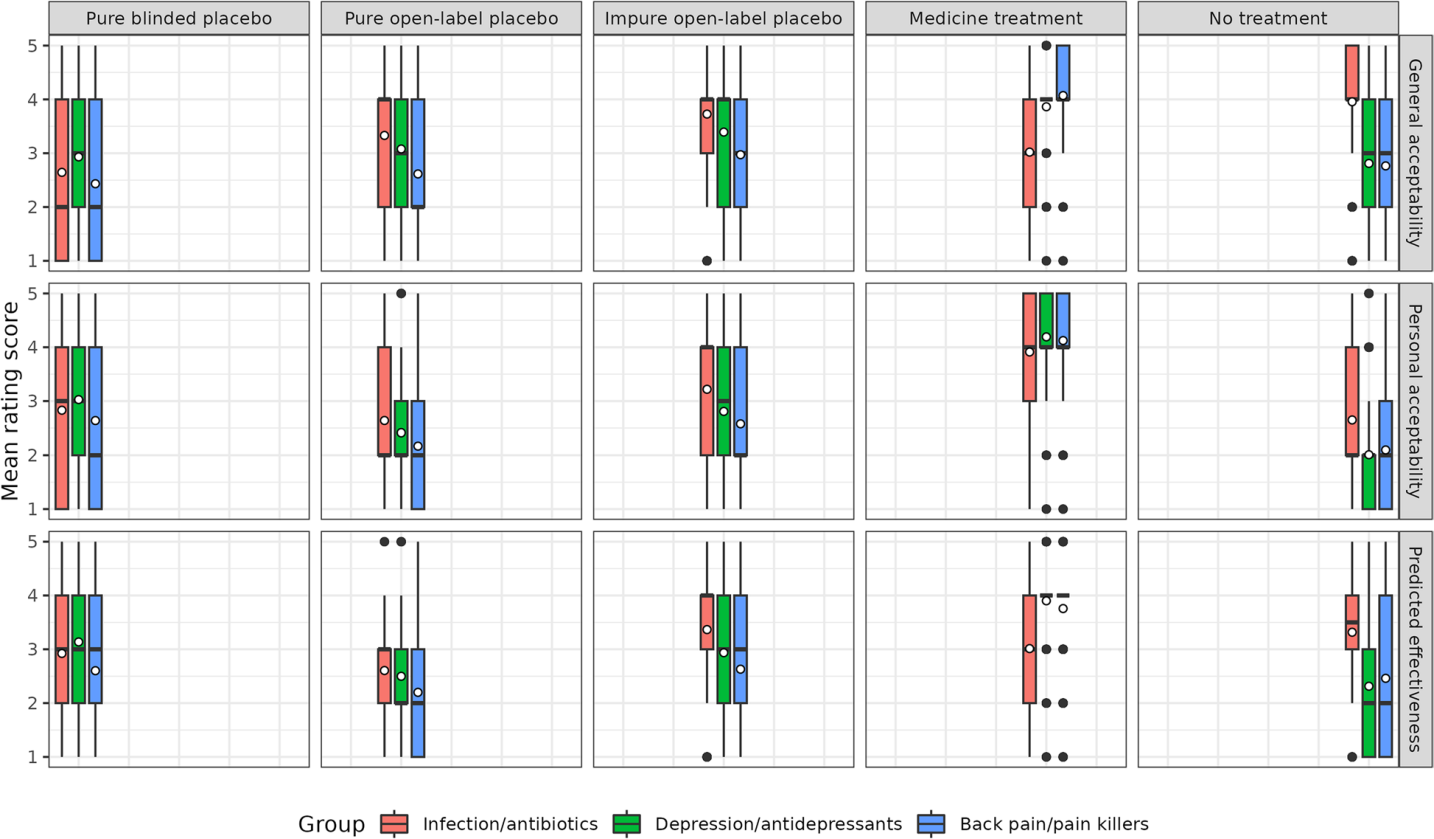
Study 3 ratings for general acceptability, personal acceptability and predicted effectiveness across five treatment conditions. Box plot showing the rating scores for general acceptability, personal acceptability and predicted effectiveness across the five treatment conditions (blinded + pure placebo; open-label + pure placebo; open-label + impure placebo; antibiotic treatment; no treatment) and three scenario groups (infection/antibiotics; depression/antidepressants; back pain/pain killers). White dots represent mean values

Three 3 × 5 mixed ANOVAs were conducted to test for effects of the scenario group (infection/antibiotics vs. depression/antidepressants vs. back pain/pain killers) and the treatment condition (blinded + pure placebo vs. open-label + pure placebo vs. open-label + impure placebo vs. antibiotic treatment vs. no treatment) on general acceptability, personal acceptability and expected effectiveness of the treatment. Descriptive statistics for all groups and conditions are presented in Additional File 1: Table 13. Figure 2 visualises the results with box plots.

For general acceptability, there was a significant interaction effect (*F*(6.99, 4105.35) = 77.420, *p* < 0.001, *η*_p_^2^ = 0.116), suggesting that the ratings for each treatment condition differed depending on the scenario group the participant had been assigned to. Contrasts showed that mean acceptability ratings for open-label pure placebos, open-label impure placebos and no treatment options were significantly higher for the infection scenario than both other scenarios. Conversely, the mean acceptability ratings for the medicine treatment option were significantly lower in the infection scenario than in both other scenarios. For the pure blinded placebo condition, mean acceptability ratings were highest for the antidepressant scenario, followed by antibiotics and pain killers. We also found a significant main effect of scenario group (*F*(2, 1175) = 27.108, *p* < 0.001, *η*_p_^2^ = 0.044). Pairwise comparisons using Bonferroni adjustments showed that general acceptability ratings across all five treatment conditions were significantly lower for the back pain scenario compared to the infection and depression scenarios. Finally, there was a significant main effect of the treatment condition, (*F*(3.494, 4105.35) = 137.032, *p* < 0.001, *η*_p_^2^ = 0.104). Pairwise comparison using Bonferroni adjustments showed that mean ratings for all treatment conditions differed significantly from one another. The “medicine” condition” received the highest ratings, and this was followed by the “open-label + impure placebo” condition, the “no treatment” condition, the “open-label + pure” condition and lastly the “blinded + pure placebo” condition.

For personal acceptability, there was a significant interaction effect (*F*(7.07, 4155.27) = 17.438, *p* < 0.001, *η*_p_^2^ = 0.029), suggesting that the ratings for each treatment condition differed depending on the scenario group the participant had been assigned to. Again, contrasts showed that mean ratings of personal acceptability for open-label pure placebos, open-label impure placebos and no treatment options were significantly higher for the infection scenario than both other scenarios. However, contrary to the ratings of general acceptability, the medicine condition and the blinded pure condition showed no significant differences based on the scenario. There was also a significant main effect of the scenario group (*F*(2, 1175) = 24.180, *p* < 0.001, *η*_p_^2^ = 0.040). Pairwise comparisons using Bonferroni adjustments showed significant differences between overall rating scores for all three scenarios, with means being highest for the infection scenario, followed by the depression scenario and lastly the back pain scenario. Finally, there was a significant main effect of the treatment condition, (*F*(3.536, 4155.27) = 524.347, *p* < 0.001, *η*_p_^2^ = 0.309). Mean ratings of personal acceptability differed significantly between each condition. They were highest for the “medicine” condition, followed by “open-label + impure placebo”, “blinded + pure placebo”, “open-label + pure placebo”, and “no treatment” conditions.

For expected effectiveness, we found a significant interaction effect (*F*(7.09, 4166.43) = 65.898, *p* < 0.001, *η*_p_^2^ = 0.101), again suggesting that the ratings for each treatment condition differed depending on the scenario group the participant had been assigned to. Contrasts showed that predicted effectiveness ratings for the open-label impure placebo condition and the no treatment condition were significantly higher for the infection scenario compared to the other two scenarios. Furthermore, mean ratings for the medicine condition were significantly lower for the infection scenario compared to the other two scenarios. There was also a significant main effect of the scenario group (*F*(2, 1175) = 27.045, *p* < 0.001, *η*_p_^2^ = 0.040). Pairwise comparisons using Bonferroni adjustments showed that the back pain scenario produced lower overall ratings of predicted effectiveness than the other two scenarios. Finally, again, there was a significant main effect of the treatment condition (*F*(3.546, 4166.428) = 215.052, *p* < 0.001, *η*_p_^2^ = 0.155). Pairwise comparisons indicated that apart from the blinded pure placebo condition and the open-label impure placebo conditions, all conditions differed significantly from one another. The “medicine” condition was rated as the most effective, followed by the “open-label + impure placebo” condition, “blinded + pure placebo”, “no treatment”, and the “open-label + pure placebo” condition.

### Brief discussion

Study 3 extended our previous focus group study and online experiment through the comparison of different prescribing contexts and their respective effects on public attitudes around placebos. We replicated our previous findings on the acceptability of different placebo types for replacing unnecessary antibiotic treatments in the context of respiratory infections and showed that public acceptability of clinical placebo use differed for different clinical scenarios.

Hence, our clinical scenario hypothesis on the effect of the prescribing context on placebo attitudes was confirmed. However, contrary to our predictions, participants demonstrated the most favourable placebo attitudes in the infection condition, followed by the depression condition and finally the back pain condition. Similar to the results from Study 2, there were notable differences between the general acceptability and the personal acceptability ratings for using antibiotics in our respiratory infection scenario. Again, participants showed a stronger preference for antibiotics when they were asked to indicate their personal willingness to receive each treatment, thus providing further evidence for differences in self and other decision-making.

The comparatively lower acceptability and efficacy ratings for clinical placebo use to treat depression and back pain are more difficult to interpret. Based on our initial focus group study, we had indeed predicted lower ratings in the context of pain management, because participants had described beliefs that highly tangible symptoms of pain may not be effectively managed with placebos. However, with several participants from the focus groups suggesting mental health as the prescribing context most appropriate for placebo use, we had expected to find more positive attitude ratings in the depression scenario. One possibility for the unexpected findings is that the infection scenario stood out by highlighting societal consequences (i.e. antimicrobial resistance) as well as personal side effects of medicine overuse. Based on this explanation, more detailed patient information on both societal and personal consequences of overtreatment across different treatment contexts might help to further raise placebo acceptability.

With regard to our second hypothesis, the Treatment type hypothesis, all our predictions were confirmed. Similar to the results from Study 2, open-label impure placebos received the highest acceptability ratings compared to the other types of placebos and the open-label pure placebo condition received the lowest ratings for expected effectiveness. Furthermore, as predicted, the active medical treatment was overall rated most favourable, even though the infection scenario differed from this pattern as described above. These findings suggest that any clinical treatment using blinded placebos should be avoided, but highlight the specific potential of using open-label impure placebo treatments such as anti-inflammatory lozenges, vitamin supplements or pain relief heat spray more strategically. Given previous survey findings on the existing practice involving the informal use of impure placebos [16], our research findings present a strong case for providing more formalised guidance on the strategic and transparent use of this placebo type.

## General discussion

Our three-part, mixed methods study provides the first comprehensive evidence for the public acceptability of clinical placebos. It thereby indicates support for a novel, behavioural approach to reduce overtreatment in primary care. Our qualitative and quantitative results both highlight particular potential for the use of open-label impure placebos, which are treatments with known pharmacological value for some of the patients’ symptoms, but without therapeutic effects for the underlying illness. This is aligned with previous research, which suggested patient support for and curiosity about open-label placebos [19–24] as part of clinical trials for drug development. Furthermore, similar to previous studies [23, 25, 26], our focus group investigation of opinions around placebo use highlighted the importance of a trusting and open doctor-patient relationship as a prerequisite for any type of clinical placebo use. Our quantitative results from Study 3 suggest that attitudes towards clinical placebo use are most favourable for cases, where overtreatment is likely to result in both personal side effects and negative consequences for wider society, such as contexts involving inappropriate antibiotic prescribing.

Our results demonstrated interesting differences in self- and other decision-making. When presented with a decision scenario on respiratory infections involving the potential inappropriate use of antibiotics, participants generally opposed the use of antibiotics. Yet, they still indicated a personal preference for receiving antibiotics if they were the ones suffering from the infection. This suggests heightened levels of loss aversion when making choices for oneself as found in previous experimental research [49, 50]. The mismatch of general and personal placebo acceptability could present a challenge when attempting to implement a roll-out of clinical placebo use. Reasons for the mismatch need to be better understood and it is likely that individual concerns about personal risks will need to be addressed, for example through effective safety-netting.

Additionally, Study 2 suggests that individual levels of placebo acceptability and beliefs around effectiveness may vary depending on a person’s demographic background, previous medical history and individual differences in health anxiety and health literacy. Older people, individuals suffering from chronic illness or those showing higher levels of health anxiety, may be less amenable to placebo use. Individuals with lower health literacy may be less accepting of open-label placebos, supporting previous research which found that health literacy predicted people’s responses to health messages [37]. Additionally, women appear to have particularly negative attitudes towards blinded placebos, which involve the use of deception. Follow-up qualitative research could be employed to shed further light on reasons behind the influences of these individual difference variables and to explore potential factors undermining trust in the context of placebo use.

Finally, our results suggest an important role of language when communicating information about placebos. Specific terms such as “impure” or “sugar pills” may carry negative connotations suggestive of contamination or child-like properties respectively, which could shape public attitudes around placebos and should therefore be avoided. These findings are aligned with previous results on the effects of language framing, including work highlighting the importance of disease names and related medical terminology [28, 29].

### Implications for clinical practice and policy

Our findings have important implications for clinical practice, because they suggest an acceptable behavioural approach towards reducing overtreatment in UK primary care. It appears that replacing unnecessary medication with open-label impure placebos could be supported by the general UK population—particularly in the context of antibiotic prescribing. Hence, our research has confirmed a key pre-requisite to conducting follow-up trials for testing the actual effectiveness of this behavioural strategy for reducing medicine overuse. Our findings could provide a starting point for informing whether placebos should feature more prominently in treatment guidelines, such as relating to infections. Whilse providing support for placebo use in clinical practice, our research raised several points that need to be considered when trialling placebo use practice.

#### Importance of shared decision-making

Our findings point to the likely importance of shared decision-making approaches when trialling clinical placebo use. A key theme emerging from our focus groups was the prerequisite of a trusting doctor-patient relationship, which is unlikely to emerge without building a rapport during a process of shared decision-making [56]. Shared decision-making could help to preserve trust in professionals, by allowing them to create a context of transparency and share detailed information about placebo options. Furthermore, open-label placebo use is unlikely to be a workable approach for every patient, as demonstrated by the significant influences of demographic variables, medical history and individual differences (e.g. health anxiety). Hence, shared decision-making could offer an important process for establishing an individualised, patient-centred plan of care. Additionally, detailed option talk could be used to discuss safety-netting and explore any personal risk concerns, which may have driven the comparatively higher acceptability of active medicine treatment when making decisions for oneself (as opposed to indicating general acceptability for the wider population).

#### Communication about placebos

When communicating about placebos, language is likely to play an important role. Both Studies 1 and 2 demonstrated public sensitivity to terminology. Our focus groups highlighted negative public associations with the term “impure”, which may affect acceptability of impure placebos. Instead, it may be favourable to describe these as “a type of placebos”, as done in Studies 2 and 3. Additionally, Study 2 suggested that using the term “sugar pill” instead of “placebo” may decrease public acceptability under certain circumstances. Additional research could help to identify further framing effects and the influences of other types of terminology that might shape public attitudes.

#### Provision of decision aids

To support the process of shared decision-making, the use of decision aids is likely going to be crucial. Both our PPI engagement activities and our focus group research highlighted the potential barrier of short appointment times for explaining the rationale behind placebo use and the scientific evidence around its effectiveness. Similarly, decision aids could provide additional background information about both personal side effects and societal consequences associated with medicine overuse in order to promote placebo acceptability. This suggestion is based on findings by Study 3, which found more favourable placebo attitudes in the infection scenario; the only scenario also highlighting societal harms of overtreatment (i.e. antimicrobial resistance).

#### Practical considerations

When designing trials of clinical placebo use, several practical considerations will need to be accounted for. This includes specific contexts for placebo use, the mitigation of adverse consequences and administrative challenges.

##### Contexts of clinical placebo use

Our initial focus group results demonstrated that participants were most supportive of placebo use for non-serious illnesses and in the context of mental health treatments (e.g. anxiety and depression). Study 3 confirmed that placebo use for less severe depression was rated more acceptable and effective than for chronic pain conditions, but attitudes were most favourable in the scenario involving inappropriate placebo use for infections. Future research will need to pay attention to the particular prescribing context and consider placebo use against the background of previous attempts to de-license ineffective or unnecessary treatments [57]. The use of placebos is intended to replace inappropriate and potentially harmful treatments rather than to introduce new treatments or increase patient demand and health-seeking behaviours (see also below). A related consideration is the definition of clinical criteria that determine whether specific patient cases qualify for placebo use. As with any form of treatment, clear guidelines will be necessary to justify a doctor’s treatment decision.

##### Mitigation of adverse consequences

Our focus groups raised a number of potential adverse consequences of clinical placebo use. These might include patients considering themselves treated through placebos and consequently taking less precaution about spreading their infections to others or failing to seek further help if symptoms worsen. An additional concern is that placebo prescribing for conditions not necessitating any medical treatment might result in an increase of health-seeking behaviours, potentially increasing patient demand for doctors’ appointments. All these consequences could undermine the overall practical utility of clinical placebo use and need to be monitored carefully in any clinical trials.

##### Administrative challenges

A final practical consideration is administrative challenges including the question about prescription charges for placebos. Our focus groups appeared divided on this point and future trials may need to collect further, context-specific data on public attitudes towards prescription charges.

### Study limitations

When interpreting our research findings, a number of theoretical and practical study limitations need to be considered.

#### Defining placebos

Previous research has highlighted the difficulties of defining what constitutes a placebo. Especially the category of “impure placebos” is not without contention, because of ambiguous terminology and inconsistent use in the past literature [58]. It could be argued that impure placebos as substances with some physiological effect on patient symptoms are part of a different approach of symptom management [59, 60]. Also, public acceptability of impure placebos could be tied to specific substances. For example, rather than having a general preference for impure placebos in the context of infection treatment, they might have a specific preference for lozenges (as opposed to nasal sprays). Additionally, at a more theoretical level, a case could be made that even active medical treatments such as antibiotics, antidepressants and pain killers could be considered placebos in the context of clinical scenarios that do not indicate their use. For example, prescribing an antibiotic for a viral infection would mean using a substance without therapeutic benefit for the illness in question. In this case, an antibiotic would be most accurately conceptualised as a placebo.

The present study used a broad definition of impure placebos and adopted a pragmatic approach, in which all active medical treatments (antibiotics, antidepressants and pain killers) were conceptualised as such without consideration of their actual appropriateness. However, it is possible for public acceptability to differ depending on the specific placebo definitions applied.

#### Use of hypothetical patient vignettes

A more practical research limitation pertained to the use of hypothetical patient scenarios across all three studies reported in this article. We asked participants to consider a fictitious patient case rather than assessing decisionmaking in real-life medical contexts. This methodological approach has many advantages such as overcoming difficulties of accessing high-quality clinical data and reaching large numbers of participants [61]. Additionally, vignettes may help to increase experimental control over the specific conditions of the scenario (e.g. specific patient symptoms and associated level of clinical uncertainty) and to ensure comparability of different scenarios (e.g. the scenarios used in Study 3). In spite of these advantages, we recognise that study vignettes limit the ecological validity of our results. It is possible that participant choices differ when considering an abstract scenario as opposed to a real-life experience. This is why future research in clinical settings will be imperative to confirm our results and draw wider conclusions about placebo use in clinical practice.

#### Attitude measurement

A final limitation is the use of Likert-type rating scales to measure attitudes around general acceptability, personal acceptability and predicted effectiveness of different treatment types. Attitudes are inherently difficult to quantify and opinions lack absolute values. While the use of Likert-type scales is common practice in attitude research and is typically associated with high internal consistency [62], it is impossible to obtain an absolute of value (e.g. an absolute risk rating) of any given topic. To reduce some of the limitations of traditional Likert scales, participants across both experimental studies were given brief descriptions for each numerical rating (e.g. 1 = highly unacceptable, 2 = somewhat unacceptable) in order to introduce more equal intervals between each score and to increase comparability between different participants’ ratings.

## Conclusions

Clinical placebo use could be a publicly acceptable approach for combatting overprescribing in primary care. Acceptability appears to be highest for the use of open-label impure placebos for cases where overtreatment is likely to result in both personal side effects and negative consequences for wider society (e.g. antibiotic overprescribing), although personal risk concerns, individual differences and co-variates will need to be considered. Clinical research is necessary to test practicalities (e.g. around doctor-patient communication and trust) and develop guidelines around clinical placebo use across a range of specific prescribing contexts.

### Supplementary Information

**Additional file 1.** Additional demographic and results tables for the studies reported in the manuscript.

**Additional file 2.** Detailed study materials for all studies reported in the manuscript.

## Data Availability

The quantitative datasets generated during the current studies, study materials and study preregistration forms are available in the Open Science Framework (OSF) with the identifier https://osf.io/k39th/.
