# Supplementary material for: Evidencing general acceptability of open-label placebo use for tackling overtreatment in primary care: a mixed methods study
Source: BMC Med. 2023 Sep 19;21:362. doi: 10.1186/s12916-023-03074-4 (PMC10510165; doi:10.1186/s12916-023-03074-4)
Supplement: Supplementary file 1 — Additional file 1. Additional demographic and results tables for the studies reported in the manuscript. [file 12916_2023_3074_MOESM1_ESM.docx]

Additional File 1

Evidencing general acceptability of open-label placebo use for tackling overtreatment in primary care: A mixed methods study

Krockow, E.M., Emerson, T., Youssef, E. Scott, S., & Tromans, S.

Table 1

*Study 1: Demographic Participant Details*

| Sex | *N*(Male) = 9 (56.25%)  *N*(Female) = 7(43.75%) |
| --- | --- |
| Age (in years) | *M* = 26.56, *SD* = 3.45, Range = 24-39 |
| Race | *N*(Black, African, Caribbean or Black British) = 13 (81.25%)  *N*(White) = 2 (12.5%)  *N*(Other) = 1 (6.25%) |
| Location within UK | *N*(Greater London) = 10 (62.5%)  *N*(Edinburghshire) = 3 (18.75%)  *N*(Cambridgeshire) = 1 (6.25%)  *N*(Kent) = 1 (6.25%)  *N*(Leicestershire) = 1 (6.25%) |
| Highest education level | *N*(University (bachelors) degree or equivalent) = 13 (81.25%)  *N*(Postgraduate degree) = 2 (12.5%)  *N*(Highschool graduate or equivalent) = 1 (6.25%) |
| Employment status | *N*(Working full time) = 8 (50%)  *N*(Working part time) = 8 (50%) |
| Prior experience with placebos | *N*(Yes – experience of participant’s friends/relations) = 7 (43.75%)  *N*(Yes – personal experience of participant) = 6 (37.5%)  *N*(No) = 3 (18.75%) |

Table 2

*Study 2: Demographic Participant Details*

| Sex | *N*(Female) = 505 (51.1%)  *N*(Male) = 470 (47.6%) |
| --- | --- |
| Age (in years) | *M* = 46.49, *SD* = 15.85, Range = 19-83 |
| Nationality | *N*(UK) = 892 (90.3%)  *N*(Other) = 88 (8.9%) |
| Race | *N*(White) = 856 (86.6%)  *N*(Asian or Asian British) = 69 (7.0%)  *N*(Black, African, Caribbean or Black British) = 28 (2.8%)  *N*(Mixed or multiple ethnic groups) = 19 (1.9%) |
| Highest education level | *N*(University (bachelors) degree or equivalent) = 384 (38.9%)  *N*(College sixth form graduate or equivalent) = 246 (24.9%)  *N*(Highschool graduate or equivalent) = 176 (17.8%)  *N*(Postgraduate degree) = 174 (17.6%) |
| Native language | *N*(English) = 900 (91.1%)  *N*(Other) = 80 (8.1%) |
| Employment status | *N*(Working full time) = 442 (44.7%)  *N*(Working part time) = 271 (22.0%)  *N*(Not working) = 231 (23.4%)  *N*(Prefer not to say) = 90 (9.1%) |
| Employment industry | *N*(Other) = 222 (22.5%)  *N*(Healthcare or social assistance) = 116 (11.7%)  *N*(Educational services) = 137 (13.9%)  *N*(Professional, scientific or technical services) = 80 (8.1%)  *N*(Retail trade) = 73 (7.4%)  *N*(Finance or insurance) = 61 (6.2%)  *N*(Manufacturing) = 49 (5.0%)  *N*(Arts, entertainment or recreation) = 43 (4.4%)  *N*(Admin, support, waste management or remediation services) = 38 (3.8%)  *N*(Information) = 37 (3.7%)  *N*(Transportation or warehousing) = 37 (3.7%)  *N*(Accommodation or food services) = 32 (3.2%)  *N*(Construction) = 23 (2.3%)  *N*(Management of companies or enterprises) = 14 (1.4%)  *N*(Real estate or rental and leasing) = 12 (1.2%)  *N*(Utilities) = 12 (1.2%)  *N*(Wholesale trade) = 7 (0.7%)  *N*(Forestry, fishing, hunting or agriculture support) = 5 (0.5%) |
| Financial dependants | *N*(No) = 691 (69.9%)  *N*(Yes) = 278 (28.1%)  *N*(Prefer not to say) = 111 (1.1%) |
| Religion | *N*(No religion) = 538 (54.5%)  *N*(Christian - all denominations) = 355 (35.9%)  *N*(Muslim) = 30 (3.0%)  *N*(Any other religion) = 23 (2.3%)  *N*(Hindu) = 9 (0.9%)  *N*(Buddhist) = 6 (0.6%)  *N*(Jewish) = 3 (0.3%)  *N*(Sikh) = 3 (0.3%) |
| Visited doctor in the past 12 months | *N*(Yes) = 681 (68.9%)  *N*(No) = 295 (29.9%)  *N*(Prefer not to say) = 4 (0.4%) |
| Taken antibiotics in the past 12 months | *N*(No) = 714 (72.3%)  *N*(Yes) = 265 (26.8%)  *N*(Prefer not to say) = 1 (0.1%) |
| Taken placebo before | *N*(No) = 953 (96.5%)  *N*(Yes) = 21 (2.1%)  *N*(Prefer not to say) = 6 (0.6%) |
| Chronic illness (e.g. diabetes) or immunocompromised (e.g. due to cancer treatment) | *N*(No) = 778 (78.7%)  *N*(Yes) = 193 (19.5%)  *N*(Prefer not to say) = 9 (0.9%) |
| Health anxiety score | *M* = 37.03, *SD* = 11.01, Range = 21-84 |
| Health literacy score | *M* = 21.73, *SD* = 3.32, Range = 11-31 |
| Health-related risk taking score | *M* = 10.77, *SD* = 3.77, Range = 6-30 |

Table 3

*Study 2: Overview of content codes and associated frequencies for the qualitative content analysis of the free-text questions*

| **Content code:** | **Freq:** | **Example data:** |
| --- | --- | --- |
| *Q1: Defining placebos* | | |
| Inert substance | 244 | *“A dummy pill. It does not contain an active ingredient”* |
| Fake / artificial medication | 132 | *“Fake drug where you think you are receiving the drug, but are in fact receiving a*  *Nothing"* |
| Deception | 97 | *“Some sort of medication/pill/substance/food that is given to someone by telling them that it makes them feel better/stronger mentally or physically even though it does not have any effects”* |
| Research / clinical trials | 80 | *“A treatment - physical, chemical or other - with no medicinal benefit, used against active treatments in medical trials.”* |
| Psychological processes | 75 | *“A drug which is given that has no chemical physiological affect. however our minds are powerful and just taking a drug can have a positive affect due to the physiological belief that the drug is helping.”* |
| *Q2: Advantages* | | |
| Positive psychological effects | 165 | *“It could potentially trigger something mentally within people that they could feel better. It would be a psychological thing and that could potentially trick the brain into making someone feel better from certain issues.”* |
| Monitoring efficacy of medications | 113 | *“It is helpful to have a control group in any research. By comparing the control and treatment groups, researchers can determine whether a specific type of medicine is truly effective.”* |
| No side effects | 69 | *“You mentally make yourself well without any medicinal side effects”* |
| Reducing use of medications | 59 | *“It would reduce the unnecessary use of medication, particularly antibiotics where antibiotic resistance can be a problem. Also reduces side effects and the need for monitoring such as blood tests.”* |
| Lower cost | 57 | *“Less toxicity, cost of treatment, fewer side effects.”* |
| Determining the nature of symptoms | 37 | *“It can diagnose whether an issue is biological or mental”* |
| Research purposes | 34 | *“It helps in research and health trials and doesn't give any bad effects on the body.”* |
| *Q3: Disadvantages* | | |
| Deception / ethical problems | 119 | *“They generally only work as long as the patient doesn't know they are a placebo”* |
| Inert / ineffective | 113 | *“A disadvantage could be that the placebo is inert and has no medical powers and the use of it will not help or assist the patient at all.”* |
| Delaying (active) treatment | 105 | *“People taking placebos will miss out on the possible benefits that the actual medication might provide”* |
| Prolonging / making illness worse | 68 | *“Some patients may feel better psychologically simply because they are taking something, when in fact their condition has not changed or worsened”* |
| Loss of trust in medicine / clinicians | 26 | *“They may work for fewer people. Not curing a problem early may cause issues in future treatment. Should a patient find out they have been treated with a placebo they may have less trust in the medical profession and may be less likely to seek help for future, unrelated illness”* |
| No disadvantages | 24 | *“No disadvantages to be honest other than the fact they are primarily for analytical purposes.”* |
| Delaying recognition of an illness | 12 | *“It is a form of deception. In some circumstances, a patient may really need the real thing and this could delay and worsen their condition.”* |
| Reliance | 11 | *“People will come to rely on them and want stronger doses”* |

Table 4

*Descriptive statistics (means and standard deviations) for each dependent variable (general acceptability, personal acceptability and expected effectiveness), presented by treatment condition and framing group.*

| Treatment condition | Framing Group | General acceptability *M* (*SD*) | Personal acceptability  *M* (*SD*) | Expected effectiveness  *M* (*SD*) |
| --- | --- | --- | --- | --- |
| Blinded + pure placebo | Placebo frame | 2.92 (1.29) | 2.95(1.39) | 2.97 (1.06) |
|  | Sugar pill frame | 2.64 (1.32) | 2.65 (1.38) | 2.81 (1.12) |
|  | Total | 2.78 (1.31) | 2.80 (1.40) | 2.89 (1.09) |
| Open-label + pure placebo | Placebo frame | 3.36 (1.20) | 2.69 (1.14) | 2.63 (1.08) |
|  | Sugar pill frame | 3.41 (1.25) | 2.65 (1.20) | 2.59 (1.08) |
|  | Total | 3.38 (1.22 | 2.67 (1.17) | 2.61 (1.08) |
| Open-label + impure placebo | Placebo frame | 3.91 (.99) | 3.41 (1.08) | 3.47 (.91) |
|  | Sugar pill frame | 3.90 (1.02) | 3.39 (1.13) | 3.46 (.96) |
|  | Total | 3.91 (1.01) | 3.40 (1.10) | 3.47 (.94) |
| Antibiotic treatment | Placebo frame | 3.16 (1.33) | 3.91 (1.16) | 3.19 (1.19) |
|  | Sugar pill frame | 3.06 (1.29) | 3.83 (1.12) | 3.12 (1.21) |
|  | Total | 3.11 (1.31) | 3.87 (1.14) | 3.16 (1.20) |
| No treatment | Placebo frame | 3.82 (1.17) | 2.60 (1.17) | 3.21 (1.08) |
|  | Sugar pill frame | 3.91 (1.07) | 2.73 (1.20) | 3.28 (1.00) |
|  | Total | 3.86 (1.12) | 2.66 (1.19) | 3.25 (1.04) |

Table 5

*Study 2: Descriptive statistics (means and standard deviations) for each dependent variable (general acceptability, personal acceptability and expected effectiveness), presented by treatment condition and binary age group*

| Treatment condition | Age (binary) | General acceptability *M* (*SD*) | Personal acceptability  *M* (*SD*) | Expected effectiveness  *M* (*SD*) |
| --- | --- | --- | --- | --- |
| Blinded + pure placebo | High (>46 years) | 2.81 (1.35) | 2.82 (1.39) | 2.72 (1.08) |
|  | Low (≤46 years) | 2.77 (1.27) | 2.79 (1.40) | 3.06 (1.08) |
|  | Total | 2.79 (1.31) | 2.80 (1.39) | 2.90 (1.09) |
| Open-label + pure placebo | High (>46 years) | 3.28 (1.24) | 2.63 (1.17) | 2.56 (1.04) |
|  | Low (≤46 years) | 3.51 (1.18) | 2.71 (1.16) | 2.66 (1.11) |
|  | Total | 3.39 (1.22) | 2.67 (1.17) | 2.61 (1.08) |
| Open-label + impure placebo | High (>46 years) | 3.90 (1.01) | 3.43 (1.10) | 3.44 (0.90) |
|  | Low (≤46 years) | 3.92 (1.01) | 3.28 (1.10) | 3.49 (0.96) |
|  | Total | 3.91 (1.01) | 3.41 (1.10) | 3.47 (0.93) |
| Antibiotic treatment | High (>46 years) | 3.05 (1.31) | 3.83 (1.17) | 3.09 (1.17) |
|  | Low (≤46 years) | 3.16 (1.30) | 3.92 (1.11) | 3.22 (1.24) |
|  | Total | 3.11 (1.31) | 3.87 (1.14) | 3.15 (1.20) |
| No treatment | High (>46 years) | 3.90 (1.12) | 2.83 (1.25) | 3.27 (1.00) |
|  | Low (≤46 years) | 3.84 (1.12) | 2.50 (1.11) | 3.23 (1.08) |
|  | Total | 3.87 (1.12) | 2.66 (1.19) | 3.25 (1.04) |

Table 6

*Study 2: Descriptive statistics (means and standard deviations) for each dependent variable (general acceptability, personal acceptability and expected effectiveness), presented by treatment condition and sex.*

| Treatment condition | Sex | General acceptability *M* (*SD*) | Personal acceptability  *M* (*SD*) | Expected effectiveness  *M* (*SD*) |
| --- | --- | --- | --- | --- |
| Blinded + pure placebo | Male | 2.99 (1.29) | 3.00 (1.40) | 2.93 (1.07) |
|  | Female | 2.59 (1.30) | 2.62 (1.36) | 2.85 (1.12) |
|  | Total | 2.78 (1.31) | 2.80 (1.40) | 2.89 (1.09) |
| Open-label + pure placebo | Male | 3.32 (1.23) | 2.59 (1.14) | 2.50 (1.06) |
|  | Female | 3.45 (1.22) | 2.75 (1.20) | 2.70 (1.09) |
|  | Total | 3.38 (1.22) | 2.67 (1.17) | 2.61 (1.08) |
| Open-label + impure placebo | Male | 3.86 (1.02) | 3.33(1.08) | 3.40 (0.96) |
|  | Female | 3.96 (0.99) | 3.47 (1.11) | 3.53 (0.90) |
|  | Total | 3.91 (1.01) | 3.40 (1.10) | 3.47 (0.93) |
| Antibiotic treatment | Male | 3.17 (1.32) | 3.96 (1.11) | 3.19 (1.20) |
|  | Female | 3.06 (1.30) | 3.79 (1.12) | 3.12 (1.19) |
|  | Total | 3.11 (1.31) | 3.87 (1.14) | 3.15 (1.20) |
| No treatment | Male | 3.84 (1.10) | 2.67 (1.18) | 3.17 (1.01) |
|  | Female | 3.88 (1.14) | 2.65 (1.19) | 3.32 (1.06) |
|  | Total | 3.86 (1.12) | 2.66 (1.19) | 3.25 (1.04) |

Table 7

*Study 2: Descriptive statistics (means and standard deviations) for each dependent variable (general acceptability, personal acceptability and expected effectiveness), presented by treatment condition and antibiotics taken in the last 12 months.*

| Treatment condition | Antibiotics taken in the last 12 months | General acceptability *M* (*SD*) | Personal acceptability  *M* (*SD*) | Expected effectiveness  *M* (*SD*) |
| --- | --- | --- | --- | --- |
| Blinded + pure placebo | Yes | 2.68 (1.24) | 2.63 (1.36) | 2.86 (1.07) |
|  | No | 2.82 (1.34) | 2.86 (1.40) | 2.90 (1.10) |
|  | Total | 2.78 (1.31) | 2.80 (1.39) | 2.89 (1.09) |
| Open-label + pure placebo | Yes | 3.20 (1.18) | 2.54 (1.12) | 2.49 (1.02) |
|  | No | 3.45 (1.23) | 2.72 (1.18) | 2.65 (1.10) |
|  | Total | 3.38 (1.22) | 2.67 (1.68) | 2.61 (1.08) |
| Open-label + impure placebo | Yes | 3.68 (1.10) | 3.17 (1.78) | 3.28 (1.02) |
|  | No | 3.99 (0.96) | 3.48 (1.06) | 3.53 (0.89) |
|  | Total | 3.91 (1.01) | 3.40 (1.10) | 3.46 (0.94) |
| Antibiotic treatment | Yes | 3.54 (1.19) | 4.03 (1.04) | 3.49 (1.16) |
|  | No | 2.95 (1.32) | 3.81 (1.17) | 3.03 (1.19) |
|  | Total | 3.11 (1.31) | 3.87 (1.14) | 3.15 (1.20) |
| No treatment | Yes | 3.68 (1.63) | 2.54 (1.13) | 3.14 (1.04) |
|  | No | 3.93 (1.10) | 2.71 (1.21) | 3.29 (1.04) |
|  | Total | 3.87 (1.12) | 2.66 (1.19) | 3.25 (1.04) |

Table 8

*Study 2: Descriptive statistics (means and standard deviations) for each dependent variable (general acceptability, personal acceptability and expected effectiveness), presented by treatment condition and self-reported chronic illness.*

| Treatment condition | Chronic illness | General acceptability *M* (*SD*) | Personal acceptability  *M* (*SD*) | Expected effectiveness  *M* (*SD*) |
| --- | --- | --- | --- | --- |
| Blinded + pure placebo | Yes | 2.66 (1.33) | 2.70 (1.39) | 2.69 (1.07) |
|  | No | 2.82 (1.31) | 2.83 (1.40) | 2.94 (1.09) |
|  | Total | 2.79 (1.31) | 2.81 (1.39) | 2.89 (1.09) |
| Open-label + pure placebo | Yes | 3.16 (1.30) | 2.49 (1.15) | 2.43 (1.07) |
|  | No | 3.44 (1.19) | 2.71 (1.16) | 2.65 (1.08) |
|  | Total | 3.39 (1.22) | 2.67 (1.17) | 2.61 (1.08) |
| Open-label + impure placebo | Yes | 3.83 (1.02) | 3.37 (1.14) | 3.42 (0.97) |
|  | No | 3.93 (1.00) | 3.41 (1.09) | 3.48 (0.93) |
|  | Total | 3.91 (1.01) | 3.40 (1.10) | 3.47 (0.94) |
| Antibiotic treatment | Yes | 3.01 (1.37) | 3.89 (1.08) | 3.17 (1.14) |
|  | No | 3.14 (1.23) | 3.88 (1.15) | 3.16 (1.22) |
|  | Total | 3.11 (1.31) | 3.88 (1.14) | 3.16 (1.20) |
| No treatment | Yes | 3.83 (1.14) | 2.83 (1.26) | 3.21 (1.01) |
|  | No | 3.87 (1.12) | 2.62 (1.17) | 3.26 (1.05) |
|  | Total | 3.86 (1.12) | 2.66 (1.19) | 3.25 (1.04) |

Table 9

*Study 2: Descriptive statistics (means and standard deviations) for each dependent variable (general acceptability, personal acceptability and expected effectiveness), presented by treatment condition and binary health anxiety score.*

| Treatment condition | Health anxiety (binary) | General acceptability *M* (*SD*) | Personal acceptability  *M* (*SD*) | Expected effectiveness  *M* (*SD*) |
| --- | --- | --- | --- | --- |
| Blinded + pure placebo | High | 2.73 (1.31) | 2.66 (1.37) | 2.85 (1.10) |
|  | Low | 2.83 (1.32) | 2.93 (1.41) | 2.93 (1.08) |
|  | Total | 2.78 (1.31) | 2.80 (1.40) | 2.89 (1.09) |
| Open-label + pure placebo | High | 3.34 (1.20) | 2.66 (1.18) | 2.58 (1.11) |
|  | Low | 3.42 (1.24) | 2.68 (1.16) | 2.63 (1.05) |
|  | Total | 3.38 (1.22) | 2.67 (1.17) | 2.61 (1.08) |
| Open-label + impure placebo | High | 3.84 (1.02) | 3.35 (1.11) | 3.43 (0.97) |
|  | Low | 3.97 (1.00) | 3.45 (1.10) | 3.49 (0.90) |
|  | Total | 3.91 (1.01) | 3.40 (1.10) | 3.47 (0.94) |
| Antibiotic treatment | High | 3.24 (1.30) | 3.93 (1.10) | 3.27 (1.22) |
|  | Low | 2.99 (1.31) | 3.82 (1.17) | 3.05 (1.17) |
|  | Total | 3.11 (1.31) | 3.87 (1.14) | 3.16 (1.20) |
| No treatment | High | 3.75 (1.17) | 2.59 (1.21) | 3.12 (1.09) |
|  | Low | 3.97 (1.07) | 2.73 (1.16) | 3.36 (0.98) |
|  | Total | 3.86 (1.12) | 2.66 (1.19) | 3.25 (1.04) |

Table 10

*Study 2: Descriptive statistics (means and standard deviations) for each dependent variable (general acceptability, personal acceptability and expected effectiveness), presented by treatment condition and binary health literacy score.*

| Treatment condition | Health literacy (binary) | General acceptability *M* (*SD*) | Personal acceptability  *M* (*SD*) | Expected effectiveness  *M* (*SD*) |
| --- | --- | --- | --- | --- |
| Blinded + pure placebo | High | 2.71 (1.36) | 2.77 (1.46) | 2.86 (1.15) |
|  | Low | 2.86 (1.26) | 2.83 (1.33) | 2.92 (1.03) |
|  | Total | 2.78 (1.31) | 2.80 (1.40) | 2.89 (1.09) |
| Open-label + pure placebo | High | 3.35 (1.27) | 2.66 (1.19) | 2.53 (1.11) |
|  | Low | 3.42 (1.18) | 2.68 (1.14) | 2.68 (1.04) |
|  | Total | 3.38 (1.22) | 2.67 (1.17) | 2.61 (1.08) |
| Open-label + impure placebo | High | 3.95 (1.03) | 3.43 (1.13) | 3.48 (0.96) |
|  | Low | 3.87 (0.99) | 3.37 (1.08) | 3.45 (0.91) |
|  | Total | 3.91 (1.01) | 3.40 (1.10) | 3.47 (0.94) |
| Antibiotic treatment | High | 3.00 (1.34) | 3.80 (1.24) | 3.09 (1.24) |
|  | Low | 3.21 (1.23) | 3.94 (1.03) | 3.22 (1.15) |
|  | Total | 3.11 (1.31) | 3.87 (1.14) | 3.16 (1.20) |
| No treatment | High | 3.92 (1.13) | 2.74 (1.24) | 3.28 (1.05) |
|  | Low | 3.81 (1.12) | 2.59 (1.12) | 3.21 (1.02) |
|  | Total | 3.86 (1.12) | 2.66 (1.19) | 3.25 (1.04) |

Table 11

*Study 2: Descriptive statistics (means and standard deviations) for each dependent variable (general acceptability, personal acceptability and expected effectiveness), presented by treatment condition and binary health risk taking score.*

| Treatment condition | Health risk taking (binary) | General acceptability *M* (*SD*) | Personal acceptability  *M* (*SD*) | Expected effectiveness  *M* (*SD*) |
| --- | --- | --- | --- | --- |
| Blinded + pure placebo | High | 2.80 (1.23) | 2.86 (1.40) | 2.92 (1.07) |
|  | Low | 2.77 (1.33) | 2.75 (1.40) | 2.86 (1.11) |
|  | Total | 2.78 (1.31) | 2.80 (1.40) | 2.89 (1.09) |
| Open-label + pure placebo | High | 3.41 (1.19) | 2.60 (1.13) | 2.51 (1.04) |
|  | Low | 3.36 (1.26) | 2.73 (1.20) | 2.69 (1.11) |
|  | Total | 3.38 (1.22) | 2.67 (1.17) | 2.61 (1.08) |
| Open-label + impure placebo | High | 3.91 (1.02) | 3.31 (1.12) | 3.41 (1.00) |
|  | Low | 3.91 (1.01) | 3.47 (1.08) | 3.51 (0.89) |
|  | Total | 3.91 (1.00) | 3.40 (1.10) | 3.47 (0.94) |
| Antibiotic treatment | High | 3.10 (1.32) | 3.94 (1.11) | 3.12 (1.24) |
|  | Low | 3.12 (1.30) | 3.81 (1.16) | 3.18 (1.16) |
|  | Total | 3.11 (1.31) | 3.87 (1.14) | 3.16 (1.20) |
| No treatment | High | 3.90 (1.09) | 2.56 (1.14) | 3.21 (1.03) |
|  | Low | 3.83 (1.15) | 2.75 (1.22) | 3.28 (1.05) |
|  | Total | 3.86 (1.12) | 2.66 (1.19) | 3.25 (1.04) |

Table 12

*Study 3: Demographic Participant Details*

| Sex | *N*(Male) = 558 (47.4%)  *N*(Female) = 616 (52.3%) |
| --- | --- |
| Age (in years) | *M* = 45.93, *SD* = 15.50, Range = 18-88 |
| Nationality | *N*(British) = 1078 (91.5%)  *N*(Other) = 100 (8.5%) |
| Race | *N*(White) = 1025 (87.0%)  *N*(Asian or Asian British) = 86 (7.3%)  *N*(Black African and Caribbean or Black British) = 25 (3.0%)  *N*(Mixed or Multiple ethnic groups) = 21 (1.8%) |
| Health anxiety score | *M* = 37.00, *SD* = 10.92, Range = 21-81 |
| Health literacy score | *M* = 25.33, *SD* = 3.27, Range = 14-33 |
| Health related risk attitudes score | *M* = 57.66, *SD* = 5.76, Range = 22-83 |
| Highest education level | *N*(University bachelor’s degree or equivalent) = 459 (39.0%)  *N*(College sixth form graduate or equivalent) = 311 (26.4%)  *N*(Postgraduate degree) = 208 (17.7%)  *N*(Highschool graduate or equivalent) = 200 (17%) |
| Employment industry | *N*(Other) = 278 (23.6%)  *N*(Educational services) = 143 (12.1%)  *N*(Health care or social assistance) = 133 (11.3%)  *N*(Professional, scientific or technical services) = 94 (8.0%)  *N*(Retail trade) = 90 (7.6%)  *N*(Information) = 73 (6.2%)  *N*(Finance or insurance) = 64 (5.4%)  *N*(Arts, entertainment or recreation) = 59 (5.0%)  *N*(Manufacturing) = 49 (4.2%)  *N*(Transportation or warehousing) = 39 (3.3%)  *N*(Admin, support, waste management or remediation services) = 37 (3.1%)  *N*(Accommodation or food services) = 32 (2.7%)  *N*(Construction) = 31 (2.6%)  *N*(Utilities) = 17 (1.4%)  *N*(Management of companies or enterprises) = 12 (1.0%)  *N*(Wholesale trade) = 12 (1.0%)  *N*(Real estate or rental and leasing) = 10 (0.8%)  *N*(Forestry, fishing, hunting or agricultural support) = 5 (0.4%) |
| Financial dependants | *N*(No) = 771 (65.4%)  *N*(Yes) = 397 (33.7%)  *N*(Prefer not to say) = 10 (0.8%) |
| Religion | *N*(no religion) = 701 (59.5%)  *N*(Christian, all denominations) = 384 (32.6%)  *N*(Muslim) = 37 (3.1%)  *N*(Any other religion) = 21 (1.8%)  *N*(Hindu) = 10 (0.8%)  *N*(Buddhist) = 4 (0.3%)  *N*(Sikh) = 4 (0.3%)  *N*(Jewish) = 3 (0.3%) |
| Visited doctor in the past 12 months | *N*(Doctor visited) = 809 (68.7%)  *N*(Doctor not visited) = 369 (31.3%) |
| Taken antibiotics in the past 12 months | *N*(No antibiotics taken) = 819 (69.5%)  *N*(Antibiotics taken) = 395 (30.5%) |
| Taken antidepressants in the past 12 months | *N* (No antidepressants taken) = 988 (83.9%)  *N* (Antidepressants taken) = 189 (16.0%)  *N* (Prefer not to say) = 1 (0.1%) |
| Taken pain killers in the past 12 months | *N*(No painkillers taken) = 932 (79.1%)  *N*(Painkillers taken) = 245 (20.8%)  *N*(Prefer not to say) = 1 (0.1%) |
| Taken placebo before | *N*(No placebo taken) = 1142 (96.9%)  *N*(Placebo taken) = 30 (2.5%)  *N*(Prefer not to say) = 6 (0.5%) |
| Chronic illness (e.g. diabetes) or immunocompromised (e.g. due to cancer treatment) | *N*(No) = 955 (81.1%)  *N*(Yes) = 212 (18.0%)  *N*(Prefer not to say) = 11 (0.9%) |

Table 13

*Descriptive statistics (means and standard deviations) for each treatment condition and scenario group in Study 3*

| Treatment condition | Scenario Group | General acceptability *M* (*SD*) | Personal acceptability  *M* (*SD*) | Expected effectiveness  *M* (*SD*) |
| --- | --- | --- | --- | --- |
| Blinded + pure placebo | Infection/antibiotics | 2.64 (1.36) | 2.83 (1.48) | 2.93(1.16) |
|  | Depression/antidepressants | 2.93 (1.32) | 3.03 (1.37) | 3.14 (1.11) |
|  | Back pain/pain killers | 2.43 (1.25) | 2.64 (1.39) | 2.60 (1.15) |
|  | Total | 2.67 (1.33) | 2.83 (1.42) | 2.89 (1.16) |
| Open-label + pure placebo | Infection/antibiotics | 3.33 (1.25) | 2.64 (1.20) | 2.61 (1.06) |
|  | Depression/antidepressants | 3.08 (1.22) | 2.42 (1.12) | 2.50 (1.07) |
|  | Back pain/pain killers | 2.62 (1.23) | 2.17 (1.11) | 2.20 (1.03) |
|  | Total | 3.01 (1.27) | 2.41 (1.16) | 2.43 (1.07) |
| Open-label + impure placebo | Infection/antibiotics | 3.73 (1.13) | 3.22 (1.13) | 3.37 (1.00) |
|  | Depression/antidepressants | 3.39 (1.16) | 2.81 (1.12) | 2.94 (1.07) |
|  | Back pain/pain killers | 2.97 (1.21) | 2.58 (1.18) | 2.63 (1.09) |
|  | Total | 3.36 (1.21) | 2.87 (1.17) | 2.98 (1.10) |
| Medicine treatment | Infection/antibiotics | 3.02 (1.28) | 3.91 (1.12) | 3.01 (1.17) |
|  | Depression/antidepressants | 3.86 (.95) | 4.19 (.87) | 3.90 (.83) |
|  | Back pain/pain killers | 4.07 (.82) | 4.12 (.85) | 3.76 (.89) |
|  | Total | 3.66 (1.13) | 4.08 (.96) | 3.56 (1.05) |
| No treatment | Infection/antibiotics | 3.96 (1.11) | 2.65 (1.20) | 3.32 (1.00.) |
|  | Depression/antidepressants | 2.81 (1.28) | 2.01 (1.06) | 2.31 (1.13) |
|  | Back pain/pain killers | 2.77 (1.29) | 2.10 (1.11) | 2.46 (1.22) |
|  | Total | 3.17 (1.35) | 2.25 (1.16) | 2.69 (1.20) |
