# Supplementary material for: Evidencing general acceptability of open-label placebo use for tackling overtreatment in primary care: a mixed methods study
Source: BMC Med. 2023 Sep 19;21:362. doi: 10.1186/s12916-023-03074-4 (PMC10510165; doi:10.1186/s12916-023-03074-4)
Supplement: Supplementary file 2 — Additional file 2. Detailed study materials for all studies reported in the manuscript. [file 12916_2023_3074_MOESM2_ESM.docx]

Additional File 2

Evidencing general acceptability of open-label placebo use for tackling overtreatment in primary care: A mixed methods study

Krockow, E.M., Emerson, T., Youssef, E. Scott, S., & Tromans, S.

# Study 1: Focus group to explore public attitudes regarding placebos

## **1. Pre-Focus Group Questionnaire**

If you consent to taking part in this study, please complete the following questions and return the questionnaire alongside the consent form. Please note that the data provided here will be anonymised and kept separately from your personal information (name and contact details).

1. Please indicate your age in years: ___________

2. Please indicate your sex:

- - - Male
    - Female
    - Other
    - Prefer Not to Say

3. Please state your nationality:________________________

4. Please state your race:

- - - White
    - Mixed or Multiple ethnic groups,
    - Asian or Asian British,
    - Black, African, Caribbean or Black British
    - Other (please specify):_________________________

5. Highest education Level:

- - - Highschool graduate or equivalent
    - College sixth form graduate or equivalent
    - University (bachelors) degree or equivalent
    - Postgraduate degree

7. What statement best describes your current employment status?

- - - working full time
    - working part time
    - not working
    - prefer not to say

8. In your own words and without consulting additional resources, how would you describe a “placebo”?

___________________________________________________________________________________________________________________________________________________________________________________________________________________________

1. Do you have any personal experiences with placebos (e.g. have you or a close relation taken a placebo in the past)? If so, please provide details.

_____________________________________________________________________________________________________________________________________________________________________________________________________________________________________________

1. Finally, do you know the meaning of any of the terms below? Please tick all that apply.

- Open-label placebo
- Pure placebo
- Impure placebo

## **2. Topic Guide for Focus Groups**

**Introduction**:

Welcome, introduction of researchers and project

Instructions regarding housekeeping:

- - Please move to a quiet location without too much background noise and stable internet connection.
  - If you disconnect from Teams during the focus group, please try to reconnect ASAP.
  - If you have any technical difficulties during the focus group please use the chat function or email the researchers to alert us to any problems.
  - The focus group will be 60-90 mins and will be recorded. Everything you say will remain confidential.
  - After the session, the entire session will be transcribed word by word. The transcript will be checked and your details removed so the document is anonymous. Once this is completed, the original recording will be deleted.

Instructions regarding focus group:

- - As you know, we have invited you to participate in this focus group because we are interested to know your views on placebos. We are interested in your views on different kinds of placebos. We would also like to ask your opinion on whether placebos could be used routinely in primary care to replace unnecessary use of medicines such as antibiotics. This is important, because many medicines are currently prescribed when they are not needed, and this can lead to avoidable side effects for patients.
  - It’s important to remember when answering and discussing questions that there are no right or wrong answers, please just be yourself and speak as honestly as possible. Also if you don’t have an answer, then that’s okay as well.
  - Please be respectful of other participants’ views and allow everybody the time to speak and voice their opinions.

Finally before we begin, are there any questions?

**Main Questions**

1. Could you please introduce yourself and tell the others why you decided to take part in this focus group today?
2. As you know, no prior knowledge is required to take part in this focus group. To bring everybody on the same page, we would like to share some general definitions. [researchers share and explain infographic on screen]. Please take a moment to reflect on the information.


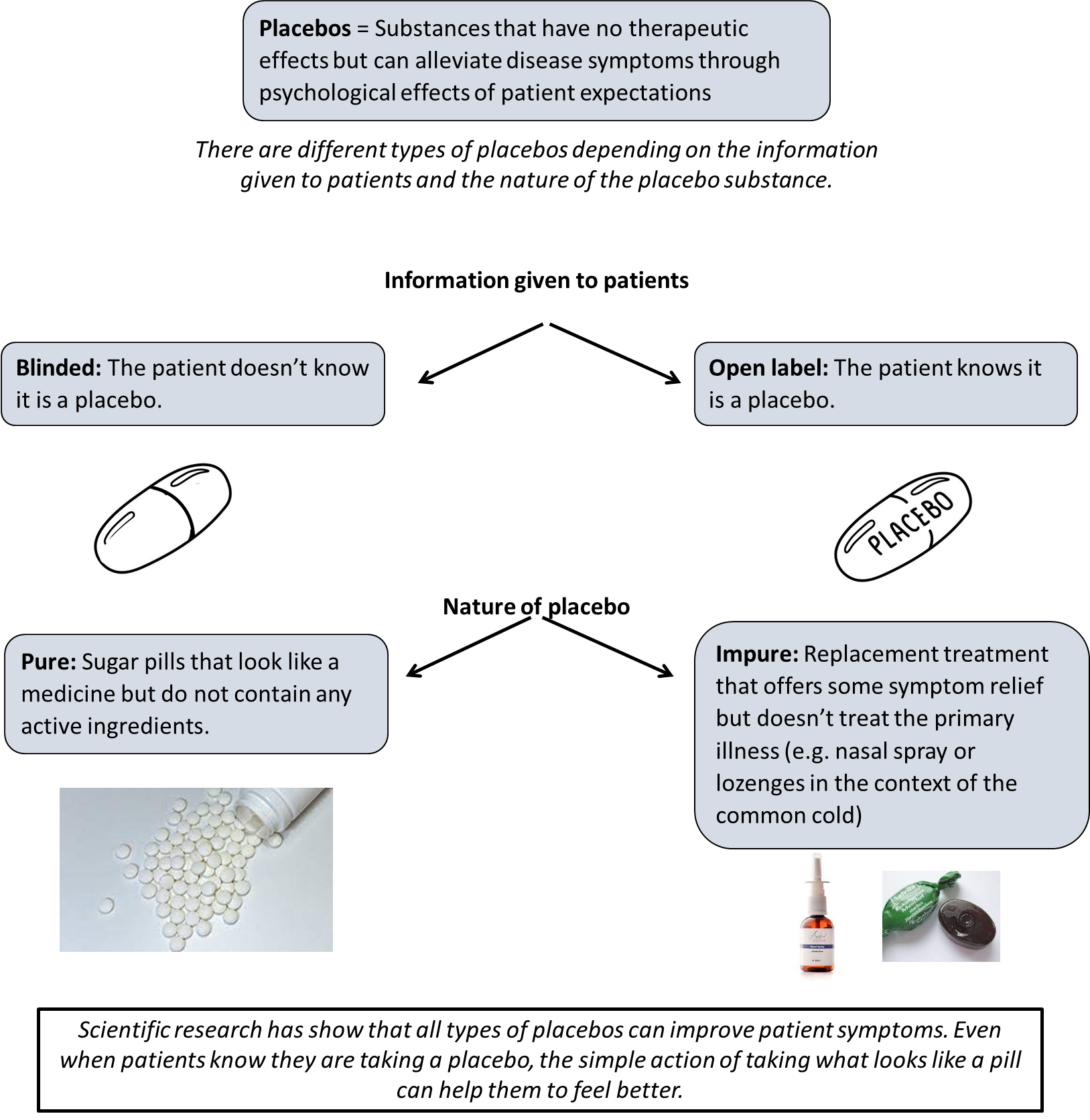


- 1. In your opinion, how easy is it to understand these different definitions?
  2. How might the definitions be improved when communicating with patients?

1. How would you describe your general opinion or attitudes about placebos?
   1. Are placebos good or bad?
   2. How do your attitudes differ across different types of placebos (blinded/open-label and pure/impure placebos)?
2. Thinking about the different types of placebos, how effective do you think these can be for symptom relief?
   1. In your opinion, which type of placebo is likely to be the most effective and why?
   2. Does it matter which medical condition placebos are prescribed for? Why yes or no?
3. Please take a look at the following hypothetical patient scenario [show scenario].

| **Scenario**  Aleksander (43 years) consults his GP for symptoms of a bad respiratory infection. He has a very sore throat, raised temperature and difficulties swallowing. Aleksander is the only breadwinner in his family of five and is keen to return to work as soon as possible, because he is on a 0-hours contract with no sick pay. He asks his GP for a prescription of antibiotics to speed up recovery.  Following a patient examination, the GP cannot be sure about the source of the infection. It is likely that Aleksander suffers from a viral infection such as the flu, for which antibiotic medication does not work. Yet, unnecessary antibiotic drugs could lead to avoidable side effects. To establish for certain whether antibiotics are needed, Aleksander would have to monitor his symptoms over the next 3-5 days.  Because of Aleksander’s preference for an active and immediate treatment, the GP offers to prescribe a placebo, i.e., a substance without therapeutic benefits, which has been shown to alleviate disease symptoms based on the power of patient expectations. If Aleksander does not get better over the next 3-5 days while taking the placebo, he is welcome to return to the GP for a review. |
| --- |

- 1. What is your opinion on offering a placebo instead of real medicine in the scenario?
  2. Should Aleksander have to pay for a prescription of placebos?
  3. What do you think are the challenges of asking patients to take placebos instead of unnecessary medicines?
  4. Do you think there are different challenges when considering the different types of placebos (i.e., blinded/open-label and pure/impure) Why yes or no?

1. Are there any other comments you would like to make regarding the scenario or placebos more generally?

**Closing statements**

- Many thanks for takin part.
- Should you think of additional comments or statements after the conclusion of today’s Focus Group, feel free to email us or give us a call.

# Study 2: Online experiment to explore public attitudes towards prescribing in primary care.

## **1. Survey questions**

*Demographics*

1. Please indicate your month and year of birth: _________________

2. Please indicate your sex:

- Male
- Female
- Other
- Prefer Not to Say

3. Please state your country of residence:

- UK

- Other (if other, please specify) :____________________

4. Please state your nationality:

- UK

- Other (if other, please specify) :____________________

5. Please state your race:

- White,
- Mixed or Multiple ethnic groups,
- Asian or Asian British,
- Black, African, Caribbean or Black British
- Other (if you selected Other, please specify):____________________

6. Highest education Level:

- Highschool graduate or equivalent
- College sixth form graduate or equivalent
- University (bachelors) degree or equivalent
- Postgraduate degree

7. Please state the name of your First/ Native Language:

- English

- Other (if other, please specify):____________________

8. If applicable, please now state any additional native languages you are fluent in:

9. What statement best describes your current employment status?

- working full time
- working part time
- not working
- prefer not to say

10. Which of the following industries most closely matches the one in which you are employed?:

- Retail trade
- Wholesale trade
- Health care or social assistance
- Professional, scientific or technical services
- Finance or insurance
- Transportation or warehousing
- Information
- Admin, support, waste management or remediation services
- Management of companies or enterprises
- Arts, entertainment or recreation
- Manufacturing
- Mining
- Forestry, fishing, hunting or agriculture support
- Real estate or rental and leasing
- Educational services
- Accommodation or food services
- Construction
- Utilities
- Other (if other, please specify):____________________

11. Do you have any person/s relying on you financially? E.g. Young children

- Yes
- No
- Prefer not to say

12. What is your religion?

- No religion
- Christian (all denominations)
- Buddhist
- Hindu
- Jewish
- Muslim
- Sikh
- Any other religion

*Medical history*

1. Have you visited a doctor in the past 12 months?

- Yes
- No
- Prefer not to say

2. Have you taken an antibiotic in the past 12 months?

- Yes
- No
- Prefer not to say

3. Have you ever taken a placebo?

- Yes
- No
- Prefer not to say

4. Do you consider yourself to have a chronic illness (e.g. diabetes) or be immunocompromised (e.g. due to cancer treatment)?

- Yes
- No
- Prefer not to say

## **2. Open-ended questions**

## **Questions for Group A (“placebo” frame)**

1. Using your own words and without consulting external resources, how would you describe the term “placebo”?

______________________________________________________________________________________________________________________________________________________

1. Based on your personal opinion, what might be some advantages associated with placebos?

______________________________________________________________________________________________________________________________________________________

1. Based on your personal opinion, what might be some disadvantages associated with placebos?

______________________________________________________________________________________________________________________________________________________

## **Questions for Group B (“sugar pill” frame)**

1. Using your own words and without consulting external resources, how would you describe the term “sugar pill”?

______________________________________________________________________________________________________________________________________________________

1. Based on your personal opinion, what might be some advantages associated with sugar pills?

______________________________________________________________________________________________________________________________________________________

1. Based on your personal opinion, what might be some disadvantages associated with sugar pills?

## **Experimental scenario**

**Please read the following hypothetical patient scenario very carefully.**

Ben (38 years) consults his GP for symptoms of a bad respiratory infection. He has a very sore throat, raised temperature and difficulties swallowing. Ben is the only breadwinner in his family of five and is keen to return to work as soon as possible, because he gets no sick pay. He asks his GP for a prescription of antibiotics to speed up recovery.

Following a patient examination, the GP cannot be sure about the source of the infection. It is most likely that Ben suffers from a viral infection such as the flu, for which antibiotic medication does not work. Yet, using unnecessary antibiotic drugs could lead to avoidable side effects. Also, any use of antibiotics increases the number of drug-resistant bacteria, meaning that antibiotics will stop working in the long run. Antibiotics should therefore only be used if absolutely necessary. To establish for certain whether antibiotics are needed, Ben would have to monitor his symptoms over the next 3-5 days.

**Below, you will be presented with different options to treat Ben’s symptoms. For each option, please rate how acceptable you find the treatment in general, how happy you would be to receive the treatment in question and how effective you expect the treatment to be.**

**Please note that there are no right or wrong answers. We are simply interested in your personal beliefs.**

1. **Treatment options for rating (one per page)**

**[Participants complete five scenarios in randomized order. Depending on the between-subjects group they’ve been assigned to, they complete the scenarios either with a “placebo” frame or with a “sugar pill” frame.]**

**Condition A: Frame = Placebo**

**1a. Treatment type: Blinded + pure placebo; Frame: Placebo**

Because of Ben’s preference for immediate treatment, the GP prescribes him a **placebo**, i.e., a substance without therapeutic benefits, which has been shown to alleviate disease symptoms based on the power of patient expectations. More specifically, scientific evidence suggests that the simple action of taking a prescription—even if it isn’t a real drug—can help people feel better. **Ben is not aware that he is receiving a placebo—he believes he is being prescribed a real antibiotic.** If Ben does not get better over the next 3-5 days while taking the **placebo**, he is welcome to return to the GP for a review.

**2a. Treatment type: Open + pure placebo; Frame: Placebo**

Because of Ben’s preference for immediate treatment, the GP offers to prescribe a **placebo**, i.e., a substance without therapeutic benefits, which has been shown to alleviate disease symptoms based on the power of patient expectations. More specifically, scientific evidence suggests that the simple action of taking a prescription—even if it isn’t a real drug—can help people feel better. **Ben is aware that he is receiving a placebo and not a real antibiotic, but the GP explains that placebos can still be effective if the patient is fully informed about the nature of their treatment.** If Ben does not get better over the next 3-5 days while taking the **placebo**, he is welcome to return to the GP for a review.

**3a. Treatment type: Open + impure placebo; Frame: Placebo**

Because of Ben’s preference for an immediate treatment, the GP offers to prescribe **a type of placebo treatment consisting of** **anti-inflammatory lozenges. This is a treatment with known pharmacological value for pain and swelling of the throat, but without therapeutic effects for the underlying infection, which caused the symptoms in the first place. However,** scientific evidence suggests that the simple action of taking a prescription—**even if it isn’t a drug for the underlying condition**—can help people feel better **overall.** **Ben is aware that he is receiving a type of placebo and not a real antibiotic.** If Ben does not get better over the next 3-5 days while taking this **type of placebo**, he is welcome to return to the GP for a review.

**4a. Treatment type: Antibiotics; Frame: N/A**

Because of Ben’s preference for immediate treatment, the GP offers to prescribe a **course of antibiotics.** If Ben does not get better over the next 3-5 days while taking the **antibiotic**, he is welcome to return to the GP for a review.

**5a. Treatment type: None; Frame: N/A**

In spite of Ben’s preference for immediate treatment, the **GP decides not to prescribe a treatment. The GP suggests Ben monitors his symptoms, while getting bed rest and drinking plenty of fluids.** If Ben does not get better over the next 3-5 days, he is welcome to return to the GP for a review.

Each scenario will be followed by the question below:

1.) From a general point of view, how acceptable do you find the treatment choice in this scenario?

(1=highly unacceptable, 2=somewhat unacceptable, 3=neither acceptable nor unacceptable, 4=somewhat acceptable, 5=highly acceptable)

2.) If you were in Ben’s situation, how happy would you be to receive the treatment described in the scenario?

(1=very unhappy, 2=somewhat unhappy, 3=neither happy nor unhappy, 4=somewhat happy, 5=very happy)

3.) How effective do you think this treatment will be in easing the symptoms described in the scenario?

(1=very ineffective, 2=somewhat ineffective, 3=neither effective nor ineffective, 4=somewhat effective, 5=very effective)

**Condition B: Frame = Sugar pill**

**1b. Placebo type: Blinded + pure; Frame: Sugar pill**

Because of Ben’s preference for immediate treatment, the GP prescribes him a **sugar pill**, i.e., a substance without therapeutic benefits, which has been shown to alleviate disease symptoms based on the power of patient expectations. More specifically, scientific evidence suggests that the simple action of taking a prescription—even if it isn’t a real drug—can help people feel better. **Ben is not aware that he is receiving a sugar pill—he believes he is being prescribed a real antibiotic.** If Ben does not get better over the next 3-5 days while taking the **sugar pill**, he is welcome to return to the GP for a review.

**2b. Placebo type: Open + pure; Frame: Sugar pill**

Because of Ben’s preference for immediate treatment, the GP offers to prescribe a **sugar pill**, i.e., a substance without therapeutic benefits, which has been shown to alleviate disease symptoms based on the power of patient expectations. More specifically, scientific evidence suggests that the simple action of taking a prescription—even if it isn’t a real drug—can help people feel better. **Ben is aware that he is receiving a sugar pill and not a real antibiotic, but the GP explains that sugar pills can still be effective if the patient is fully informed about the nature of their treatment.** If Ben does not get better over the next 3-5 days while taking the **sugar pill**, he is welcome to return to the GP for a review.

**3b. Placebo type: Open + impure; Frame: Sugar pill**

Because of Ben’s preference for an immediate treatment, the GP offers to prescribe **a type of sugar pill treatment consisting of** **anti-inflammatory lozenges. This is a treatment with known pharmacological value for pain and swelling of the throat, but without therapeutic effects for the underlying infection, which caused the symptoms in the first place. However,** scientific evidence suggests that the simple action of taking a prescription—**even if it isn’t a drug for the underlying condition**—can help people feel better **overall.** **Ben is aware that he is receiving a type of sugar pill and not a real antibiotic.** If Ben does not get better over the next 3-5 days while taking this **type of sugar pill**, he is welcome to return to the GP for a review.

**4b. Treatment type: Antibiotics; Frame: N/A**

Because of Ben’s preference for immediate treatment, the GP offers to prescribe a **course of antibiotics.** If Ben does not get better over the next 3-5 days while taking the **antibiotic**, he is welcome to return to the GP for a review.

**5b. Treatment type: None; Frame: N/A**

In spite of Ben’s preference for immediate treatment, the **GP decides not to prescribe a treatment. The GP suggests Ben monitors his symptoms, while getting bed rest and drinking plenty of fluids.** If Ben does not get better over the next 3-5 days, he is welcome to return to the GP for a review.

Each scenario will be followed by the question below:

1.) From a general point of view, how acceptable do you find the treatment choice in this scenario?

(1=highly unacceptable, 2=somewhat unacceptable, 3=neither acceptable nor unacceptable, 4=somewhat acceptable, 5=highly acceptable)

2.) If you were in Ben’s situation, how happy would you be to receive the treatment described in the scenario?

(1=very unhappy, 2=somewhat unhappy, 3=neither happy nor unhappy, 4=somewhat happy, 5=very happy)

3.) How effective do you think this treatment will be in easing the symptoms described in the scenario?

(1=very ineffective, 2=somewhat ineffective, 3=neither effective nor ineffective, 4=somewhat effective, 5=very effective)

## **5. Health Anxiety Questionnaire**

Please read the questions below and rate your response from 1-4,

1= ‘not at all or rarely,’

2 = ‘sometimes,’

3= ‘often’

4= ‘most of the time’

1. Do you ever worry about your health?

2. Are you ever worried that you may get a serious illness in the future?

3. Does the thought of a serious illness ever scare you?

4. When you notice an unpleasant feeling in your body, do you tend to find it difficult to think of anything else?

5. Do you ever examine your body to find whether there is something wrong?

6. If you have an ache or pain do you worry that it may be caused by a serious illness?

7. Do you ever find it difficult to keep worries about your health out of your mind?

8. When you notice an unpleasant feeling in your body, do you ever worry about it?

9. When you wake up in the morning do you find you very soon begin to worry about your health?

10. When you hear of a serious illness or the death of someone you know, does it ever make you more concerned about your own health?

11. When you read or hear about an illness on TV or radio does it ever make you think you may be suffering from that illness?

12. When you experience unpleasant feelings in your body do you tend to ask friends or family about them?

13. Do you tend to read up about illness and diseases to see if you may be suffering from one?

14. Do you ever feel afraid of news that reminds you of death (such as funerals, obituary notices)?

15. Do you ever feel afraid that you may die soon?

16. Do you ever feel afraid that you may have cancer?

17. Do you ever feel afraid that you might have heart disease?

18. Do you ever feel afraid that you may have any other serious illness?

19. Have your bodily symptoms stopped you from working during the past six months or so?

20. Do your bodily symptoms stop you from concentrating on what you are doing?

21. Do your bodily symptoms stop you from enjoying yourself?

## **6. Health Literacy Questionnaire**

Please read the questions below and choose the most appropriate response (rarely, sometimes or often).

1. How often do you need someone to help you when you are given information to read by your doctor, nurse, or pharmacist?
2. When you need help, can you easily get hold of someone to assist you?
3. Do you need help to fill in official documents?
4. When you talk to a doctor or nurse, do you give them all the information they need to help you?
5. When you talk to a doctor or nurse, do you ask the questions you need to ask?
6. When you talk to a doctor or nurse, do you make sure they explain anything that you do not understand?
7. Are you someone who likes to find out lots of different information about your health?
8. How often do you think carefully about whether health information makes sense in your particular situation?
9. How often do you try to work out whether information about your health can be trusted?
10. Are you the sort of person who might question your doctor or nurse’s advice based on your own research?
11. Do you think that there plenty of ways to have a say in what the government does about health?

## **7. DOSPERT Risk-Taking Scale (Health/Safety Sub-Scale)**

For each of the following statements, please indicate the likelihood that you would engage in the described activity or behavior if you were to find yourself in that situation. Provide a rating from Extremely Unlikely to Extremely Likely.

1. Drinking heavily at a social function.
2. Engaging in unprotected sex.
3. Driving a car without wearing a seat belt.
4. Riding a motorcycle without a helmet.
5. Sunbathing without sunscreen.
6. Walking home alone at night in an unsafe area of town.

**8. Study Debrief**

The research is being conducted to investigate how people think about different types of placebo prescriptions. Specifically, we are interested in finding out whether using placebos could be an approach to avoid the use of unnecessary antibiotic medication in primary care.

This study looks at a number of different factors that might influence how people think about placebos. Firstly, we investigate whether the terminology used to describe placebos matters. We do this by comparing the terms “placebo” and “sugar pill” and test whether the different terms affect people’s attitudes. We are also interested in how people think about different types of placebos. This includes, amongst others, traditional blinded placebos where patients don’t know they are receiving a placebo, and so-called open-label placebos, where patients are informed they are receiving a placebo.

Finally, we look whether attitudes around placebos differ for different demographic groups or participants, and whether they may be predicted by concepts such as health anxiety, health literacy and risk-taking attitudes.

The overall aim is to identify ways of reducing antibiotic overuse and tackling the health threat of antimicrobial resistance.

For more information about Antimicrobial resistance, please visit the following links:

World Health Organisation: https://www.who.int/health-topics/antimicrobial-resistance.

# Study 3: Online Study to explore public attitudes towards prescribing in primary care

## **1 Demographic questions**

1. Please indicate your month and year of birth: __________________

2. Please indicate your sex:

- Male
- Female
- Other
- Prefer Not to Say

3. Please state your country of residence:

- UK

- Other (if other, please specify): ___________________

4. Please state your nationality:

- UK

- Other (if other, please specify): ___________________

5. Please state your race:

- White,
- Mixed or Multiple ethnic groups,
- Asian or Asian British,
- Black, African, Caribbean or Black British
- Other (if other, please specify): ___________________

6. Highest education Level:

- Highschool graduate or equivalent
- College sixth form graduate or equivalent
- University (bachelors) degree or equivalent
- Postgraduate degree

9. What statement best describes your current employment status?

- working full time
- working part time
- not working
- prefer not to say

10. Which of the following industries most closely matches the one in which you are employed?:

- Retail trade
- Wholesale trade
- Health care or social assistance
- Professional, scientific or technical services
- Finance or insurance
- Transportation or warehousing
- Information
- Admin, support, waste management or remediation services
- Management of companies or enterprises
- Arts, entertainment or recreation
- Manufacturing
- Mining
- Forestry, fishing, hunting or agriculture support
- Real estate or rental and leasing
- Educational services
- Accommodation or food services
- Construction
- Utilities
- Other (if other, please specify): ___________________

11. Do you have any person/s relying on you financially? E.g. Young children

- Yes
- No
- Prefer not to say

12. What is your religion?

- No religion
- Christian (all denominations)
- Buddhist
- Hindu
- Jewish
- Muslim
- Sikh
- Any other religion
- Prefer not to say

**Medical History**

1. Have you visited a doctor in the past 12 months?

- Yes
- No
- Prefer not to say

2. Have you taken an antibiotic in the past 12 months?

- Yes
- No
- Prefer not to say

3. Have you taken an antidepressant in the past 12 months?

- Yes
- No
- Prefer not to say

4. Have you taken any prescription pain killers in the past 12 months?

- Yes
- No
- Prefer not to say

5. Have you ever taken a placebo?

- Yes
- No
- Prefer not to say

6. Do you consider yourself to have a chronic illness (e.g. diabetes) or be immunocompromised (e.g. due to cancer treatment)?

- Yes

- No

- Prefer not to say

## **2. Experimental scenarios**

[Participants are randomly assigned to one of three groups (A, B or C). Each group will receive a different experimental scenario.]

**Group A = Antibiotics**

**Please read the following hypothetical patient scenario very carefully.**

Ben (38 years) consults his GP for symptoms of a respiratory infection. He has a very sore throat, raised temperature and difficulties swallowing. Ben is the only breadwinner in his family of five and is keen to return to work as soon as possible, because he gets no sick pay. He asks his GP for a prescription of antibiotics to speed up recovery.

Following a patient examination, the GP cannot be sure about the source of the infection. It is most likely that Ben suffers from a viral infection such as the flu, for which antibiotic medication does not work. Yet, using unnecessary antibiotic drugs could lead to avoidable side effects. Also, any use of antibiotics increases the number of drug-resistant bacteria, meaning that antibiotics will stop working in the long run. Antibiotics should therefore only be used if absolutely necessary. To establish for certain whether antibiotics are needed, Ben would have to monitor his symptoms over the next 3-5 days.

**Below, you will be presented with different options to treat Ben’s symptoms. For each option, please rate how acceptable you find the treatment in general, how happy you would be to receive the treatment in question and how effective you expect the treatment to be.**

**Please note that there are no right or wrong answers. We are simply interested in your personal beliefs.**

**Group B = Antidepressants**

**Please read the following hypothetical patient scenario very carefully.**

Ben (38 years) consults his GP for symptoms of depression. He often feels low and hopeless about the future most of the time. He also experiences low self-worth, reduced levels of energy and constantly feels like sleeping. Ben is the only breadwinner in his family of five and the symptoms are interfering with his work, resulting in recurrent periods of absence. Ben is keen to return to work as soon as possible, because he gets no sick pay. He asks his GP for a prescription of antidepressants to speed up recovery.

Following a patient examination, the GP diagnoses less severe depression, for which antidepressant medication is not always given. Like any medication, using unnecessary antidepressants could lead to otherwise avoidable side effects. Furthermore, alternative treatments such as psychological therapies are available. Consequently, the GP puts Ben on the waiting list for psychological therapy, but there are different options for how to manage the symptoms while waiting for a therapy space.

**Below, you will be presented with different options to treat Ben’s symptoms. For each option, please rate how acceptable you find the treatment in general, how happy you would be to receive the treatment in question and how effective you expect the treatment to be.**

**Please note that there are no right or wrong answers. We are simply interested in your personal beliefs.**

**Group C = Pain medication**

**Please read the following hypothetical patient scenario very carefully.**

Ben (38 years) consults his GP for lower back pain. His lower back feels stiff and he sometimes gets a sharp pain in his buttocks travelling down his leg to his foot, especially when bending forward. Ben is the only breadwinner in his family of five and the symptoms are interfering with his work, resulting in recurrent periods of absence. Ben is keen to return to work as soon as possible, because he gets no sick pay. He asks his GP for a prescription of strong pain killers to speed up recovery.

Following a patient examination, the GP tells Ben that the mostly likely cause of his pain is a slipped disc in his lower back, which irritates a nerve in his back. The GP tells Ben that the best treatment for a slipped disc is physiotherapy. Pain killers don’t help to heal a slipped disc and whilst some people get relief with pain killers, others get no benefit at all. Like any medication, using pain killers can lead to otherwise avoidable side effects. Consequently, the GP puts Ben on the waiting list for physiotherapy therapy, but there are different options for how to manage the symptoms while waiting for a therapy space.

**Below, you will be presented with different options to treat Ben’s symptoms. For each option, please rate how acceptable you find the treatment in general, how happy you would be to receive the treatment in question and how effective you expect the treatment to be.**

**Please note that there are no right or wrong answers. We are simply interested in your personal beliefs.**

1. **Treatment options for rating (one per page)**

**[Participants complete five scenarios in randomized order. Depending on the between-subjects group they’ve been assigned to, they complete the scenarios for Group A, B or C.]**

**Group A = Antibiotics**

**1a. Treatment type: Blinded + pure placebo; Frame: Antibiotics**

Because of Ben’s preference for immediate treatment with medication, the GP prescribes him a **placebo**, i.e., a substance without therapeutic benefits, which has been shown to alleviate disease symptoms based on the power of patient expectations. More specifically, scientific evidence suggests that the simple action of taking a prescription—even if it isn’t a real drug—can help people feel better. **Ben is not aware that he is receiving a placebo—he believes he is being prescribed a real antibiotic.** If Ben does not get better over the **next 3-5 days** while taking the **placebo**, he is welcome to return to the GP for a review.

**2a. Treatment type: Open + pure placebo; Frame: Antibiotics**

Because of Ben’s preference for immediate treatment with medication, the GP offers to prescribe a **placebo**, i.e., a substance without therapeutic benefits, which has been shown to alleviate disease symptoms based on the power of patient expectations. More specifically, scientific evidence suggests that the simple action of taking a prescription—even if it isn’t a real drug—can help people feel better. **Ben is aware that he is receiving a placebo and not a real antibiotic, but the GP explains that placebos can still be effective if the patient is fully informed about the nature of their treatment.** If Ben does not get better over the **next 3-5 days** while taking the **placebo**, he is welcome to return to the GP for a review.

**3a. Treatment type: Open + impure placebo; Frame: Antibiotics**

Because of Ben’s preference for an immediate treatment with medication, the GP offers to prescribe **a type of placebo treatment consisting of** **anti-inflammatory lozenges. This is a treatment with known pharmacological value for pain and swelling of the throat, but without therapeutic effects for the underlying infection, which caused the symptoms in the first place. However,** scientific evidence suggests that the simple action of taking a prescription—**even if it isn’t a drug for the underlying condition**—can help people feel better **overall.** **Ben is aware that he is receiving a type of placebo and not a real antibiotic.** If Ben does not get better over the **next 3-5 days** while taking this **type of placebo**, he is welcome to return to the GP for a review.

**4a. Treatment type: Drug requested by patient; Frame: Antibiotics**

Because of Ben’s preference for immediate treatment with medication, the GP offers to prescribe a **course of antibiotics.** If Ben does not get better over the **next 3-5 days** while taking the **antibiotic**, he is welcome to return to the GP for a review.

**5a. Treatment type: None; Frame: Antibiotics**

In spite of Ben’s preference for immediate treatment with medication, the **GP decides not to prescribe a treatment. The GP suggests Ben monitors his symptoms, while getting bed rest and drinking plenty of fluids.** If Ben does not get better over the **next 3-5 days**, he is welcome to return to the GP for a review.

Each scenario will be followed by the question below:

1.) From a general point of view, how acceptable do you find the treatment choice in this scenario?

(1=highly unacceptable, 2=somewhat unacceptable, 3=neither acceptable nor unacceptable, 4=somewhat acceptable, 5=highly acceptable)

2.) If you were in Ben’s situation, how happy would you be to receive the treatment described in the scenario?

(1=very unhappy, 2=somewhat unhappy, 3=neither happy nor unhappy, 4=somewhat happy, 5=very happy)

3.) How effective do you think this treatment will be in easing the symptoms described in the scenario?

(1=very ineffective, 2=somewhat ineffective, 3=neither effective nor ineffective, 4=somewhat effective, 5=very effective)

**Group B: Antidepressants**

**1b. Treatment type: Blinded + pure placebo; Frame: Antidepressants**

Because of Ben’s preference for immediate treatment with medication, the GP prescribes him a **placebo**, i.e., a substance without therapeutic benefits, which has been shown to alleviate disease symptoms based on the power of patient expectations. More specifically, scientific evidence suggests that the simple action of taking a prescription—even if it isn’t a real drug—can help people feel better. **Ben is not aware that he is receiving a placebo—he believes he is being prescribed a real antidepressant.** **Ben will be reviewed again by his GP in two to four weeks’ time.**

**2b. Treatment type: Open + pure placebo; Frame: Antidepressants**

Because of Ben’s preference for immediate treatment with medication, the GP offers to prescribe a **placebo**, i.e., a substance without therapeutic benefits, which has been shown to alleviate disease symptoms based on the power of patient expectations. More specifically, scientific evidence suggests that the simple action of taking a prescription—even if it isn’t a real drug—can help people feel better. **Ben is aware that he is receiving a placebo and not a real antidepressant, but the GP explains that placebos can still be effective if the patient is fully informed about the nature of their treatment.** **Ben will be reviewed again by his GP in two to four weeks’ time.**

**3b. Treatment type: Open + impure placebo; Frame: Antidepressants**

Because of Ben’s preference for an immediate treatment with medication, the GP offers to prescribe **a type of placebo treatment consisting of** **Vitamin B12 pills. This is a treatment with known pharmacological value for fighting fatigue, but without therapeutic effects for the underlying depression, which caused the symptoms in the first place. However,** scientific evidence suggests that the simple action of taking a prescription—**even if it isn’t a drug for the underlying condition**—can help people feel better **overall.** **Ben is aware that he is receiving a type of placebo and not a real antidepressant.** **Ben will be reviewed again by his GP in two to four weeks’ time.**

**4b. Treatment type: Drug requested by patient; Frame: Antidepressants**

Because of Ben’s preference for immediate treatment with medication, the GP offers to prescribe a **course of antidepressants. Ben will be reviewed again by his GP in two to four weeks’ time.**

**5b. Treatment type: None; Frame: Antidepressants**

In spite of Ben’s preference for immediate treatment with medication, the **GP decides not to prescribe a treatment. The GP suggests Ben monitors his symptoms, while following guided self-help.** **Ben will be reviewed again by his GP in two to four weeks’ time.**

Each scenario will be followed by the question below:

1.) From a general point of view, how acceptable do you find the treatment choice in this scenario?

(1=highly unacceptable, 2=somewhat unacceptable, 3=neither acceptable nor unacceptable, 4=somewhat acceptable, 5=highly acceptable)

2.) If you were in Ben’s situation, how happy would you be to receive the treatment described in the scenario?

(1=very unhappy, 2=somewhat unhappy, 3=neither happy nor unhappy, 4=somewhat happy, 5=very happy)

3.) How effective do you think this treatment will be in easing the symptoms described in the scenario?

(1=very ineffective, 2=somewhat ineffective, 3=neither effective nor ineffective, 4=somewhat effective, 5=very effective)

**Group C: Pain medication**

**1c. Treatment type: Blinded + pure placebo; Frame: Pain medication**

Because of Ben’s preference for immediate treatment with medication, the GP prescribes him a **placebo**, i.e., a substance without therapeutic benefits, which has been shown to alleviate disease symptoms based on the power of patient expectations. More specifically, scientific evidence suggests that the simple action of taking a prescription—even if it isn’t a real drug—can help people feel better. **Ben is not aware that he is receiving a placebo—he believes he is being prescribed a real pain killer.** **Ben will be reviewed again by his GP in two to four weeks’ time.**

**2c. Treatment type: Open + pure placebo; Frame: Pain medication**

Because of Ben’s preference for immediate treatment with medication, the GP offers to prescribe a **placebo**, i.e., a substance without therapeutic benefits, which has been shown to alleviate disease symptoms based on the power of patient expectations. More specifically, scientific evidence suggests that the simple action of taking a prescription—even if it isn’t a real drug—can help people feel better. **Ben is aware that he is receiving a placebo and not a real pain killer, but the GP explains that placebos can still be effective if the patient is fully informed about the nature of their treatment.** **Ben will be reviewed again by his GP in two to four weeks’ time.**

**3c. Treatment type: Open + impure placebo; Frame: Pain medication**

Because of Ben’s preference for an immediate treatment with medication, the GP offers to prescribe **a type of placebo treatment consisting of** **pain relief heat spray. This is a treatment that provides warming relief for back pain, but without therapeutic effects for irritated nerves from the slipped disk, which caused the symptoms in the first place. However,** scientific evidence suggests that the simple action of taking a prescription—**even if it isn’t a drug for the underlying condition**—can help people feel better **overall.** **Ben is aware that he is receiving a type of placebo and not a real pain killer.** **Ben will be reviewed again by his GP in two to four weeks’ time.**

**4c. Treatment type: Drug requested by patient; Frame: Pain medication**

Because of Ben’s preference for immediate treatment with medication, the GP offers to prescribe a **pain killer. Ben will be reviewed again by his GP in two to four weeks’ time.**

**5c. Treatment type: None; Frame: Pain medication**

In spite of Ben’s preference for immediate treatment with medication, the **GP decides not to prescribe a treatment. The GP suggests Ben monitors his symptoms, while engaging in gentle exercise.** **Ben will be reviewed again by his GP in two to four weeks’ time.**

Each scenario will be followed by the question below:

1.) From a general point of view, how acceptable do you find the treatment choice in this scenario?

(1=highly unacceptable, 2=somewhat unacceptable, 3=neither acceptable nor unacceptable, 4=somewhat acceptable, 5=highly acceptable)

2.) If you were in Ben’s situation, how happy would you be to receive the treatment described in the scenario?

(1=very unhappy, 2=somewhat unhappy, 3=neither happy nor unhappy, 4=somewhat happy, 5=very happy)

3.) How effective do you think this treatment will be in easing the symptoms described in the scenario?

(1=very ineffective, 2=somewhat ineffective, 3=neither effective nor ineffective, 4=somewhat effective, 5=very effective)

## **4. Health Anxiety Questionnaire**

Please read the questions below and rate your response from 1-4,

1= ‘not at all or rarely,’

2 = ‘sometimes,’

3= ‘often’

4= ‘most of the time’

1. Do you ever worry about your health?

2. Are you ever worried that you may get a serious illness in the future?

3. Does the thought of a serious illness ever scare you?

4. When you notice an unpleasant feeling in your body, do you tend to find it difficult to think of anything else?

5. Do you ever examine your body to find whether there is something wrong?

6. If you have an ache or pain do you worry that it may be caused by a serious illness?

7. Do you ever find it difficult to keep worries about your health out of your mind?

8. When you notice an unpleasant feeling in your body, do you ever worry about it?

9. When you wake up in the morning do you find you very soon begin to worry about your health?

10. When you hear of a serious illness or the death of someone you know, does it ever make you more concerned about your own health?

11. When you read or hear about an illness on TV or radio does it ever make you think you may be suffering from that illness?

12. When you experience unpleasant feelings in your body do you tend to ask friends or family about them?

13. Do you tend to read up about illness and diseases to see if you may be suffering from one?

14. Do you ever feel afraid of news that reminds you of death (such as funerals, obituary notices)?

15. Do you ever feel afraid that you may die soon?

16. Do you ever feel afraid that you may have cancer?

17. Do you ever feel afraid that you might have heart disease?

18. Do you ever feel afraid that you may have any other serious illness?

19. Have your bodily symptoms stopped you from working during the past six months or so?

20. Do your bodily symptoms stop you from concentrating on what you are doing?

21. Do your bodily symptoms stop you from enjoying yourself?

## **5. Health Literacy Questionnaire**

Please read the questions below and choose the most appropriate response (rarely, sometimes or often).

1. How often do you need someone to help you when you are given information to read by your doctor, nurse, or pharmacist?
2. When you need help, can you easily get hold of someone to assist you?
3. Do you need help to fill in official documents?
4. When you talk to a doctor or nurse, do you give them all the information they need to help you?
5. When you talk to a doctor or nurse, do you ask the questions you need to ask?
6. When you talk to a doctor or nurse, do you make sure they explain anything that you do not understand?
7. Are you someone who likes to find out lots of different information about your health?
8. How often do you think carefully about whether health information makes sense in your particular situation?
9. How often do you try to work out whether information about your health can be trusted?
10. Are you the sort of person who might question your doctor or nurse’s advice based on your own research?
11. Do you think that there plenty of ways to have a say in what the government does about health?

## **6. Health-Risk Attitude Scale (HRAS-13)**

Please read the questions below and rate your response from 1= ‘completely disagree’ to 7 = ‘completely agree’.

1. I think that I take good care of my body.
2. I don't want to have to consider the consequences for my health in everything that I do.
3. It is important for me to organise my life in a way that will benefit my health later on.
4. When it comes to my health, I consider myself a risk avoider.
5. My health means everything to me.
6. Looking back at my past, I would say that I have generally taken risks with my health.
7. I don’t worry too much about my health in what I do.
8. Uncertainty about the consequences of a medical intervention is, in general, part of the deal.
9. Safety has priority where my health is concerned.
10. To ensure good health now and later, I am prepared to forego a lot of things.
11. People say that I take risks with my health because of my habits.
12. If the doctor can’t give me assurances about the possible consequences of a medical intervention, then I’d rather not have it.
13. In general, I would estimate that I would not have much of a problem with undergoing a high risk operation.

**7. Study Debrief**

Thank you for taking the time to complete this study.

The research is being conducted to investigate how people think about different types of placebo prescriptions. Specifically, we are interested in finding out whether using placebos could be an approach to avoid the use of unnecessary medication in primary care (e.g. unnecessary use of antibiotics, antidepressants or pain killers).

This study looks at a number of different factors that might influence how people think about placebos. Firstly, we compared attitudes towards different types of placebos including traditional blinded placebos where patients don’t know they are receiving a placebo, and so-called “open-label placebos”, where patients are informed they are receiving a placebo.

We also test whether attitudes around placebos differ for different demographic groups of participants, and whether they may be predicted by concepts such as health anxiety, health literacy and risk-taking attitudes.

The overall aim is to identify ways of reducing unnecessary use of medicines and thereby minimising unnecessary side effects to patients.
